# Immune biomarkers link air pollution exposure to blood pressure in adolescents

**DOI:** 10.1186/s12940-020-00662-2

**Published:** 2020-10-16

**Authors:** Mary Prunicki, Nicholas Cauwenberghs, Jennifer Arthur Ataam, Hesam Movassagh, Juyong Brian Kim, Tatiana Kuznetsova, Joseph C. Wu, Holden Maecker, Francois Haddad, Kari Nadeau

**Affiliations:** 1 Sean N Parker Center for Allergy and Asthma Research, Stanford University, Stanford, USA; 2 Research Unit Hypertension and Cardiovascular Epidemiology, KU Leuven Department of Cardiovascular Sciences, University of Leuven, Leuven, Belgium; 3 Research and Innovation Unit, INSERM U999, DHU TORINO, Paris Sud University, Marie Lannelongue Hospital, Le Plessis Robinson, France; 4 Institute for Immunity, Transplantation, and Infection, Stanford University, Stanford, USA; 5 Division of Cardiovascular Medicine, Department of Medicine, Stanford University, Stanford, USA; 6 Stanford Cardiovascular Institute, Stanford University School of Medicine, Stanford, USA

**Keywords:** Adolescent, Blood pressure, Immune, Inflammation, Air pollution, Cardiovascular disease

## Abstract

**Abstract:**

**Background:**

Childhood exposure to air pollution contributes to cardiovascular disease in adulthood. Immune and oxidative stress disturbances might mediate the effects of air pollution on the cardiovascular system, but the underlying mechanisms are poorly understood in adolescents. Therefore, we aimed to identify immune biomarkers linking air pollution exposure and blood pressure levels in adolescents.

**Methods:**

We randomly recruited 100 adolescents (mean age, 16 years) from Fresno, California. Using central-site data, spatial-temporal modeling, and distance weighting exposures to the participant’s home, we estimated average pollutant levels [particulate matter (PM), polyaromatic hydrocarbons (PAH), ozone (O_3_), carbon monoxide (CO) and nitrogen oxides (NO_x_)]. We collected blood samples and vital signs on health visits. Using proteomic platforms, we quantitated markers of inflammation, oxidative stress, coagulation, and endothelial function. Immune cellular characterization was performed via mass cytometry (CyTOF). We investigated associations between pollutant levels, cytokines, immune cell types, and blood pressure (BP) using partial least squares (PLS) and linear regression, while adjusting for important confounders.

**Results:**

Using PLS, biomarkers explaining most of the variance in air pollution exposure included markers of oxidative stress (GDF-15 and myeloperoxidase), acute inflammation (C-reactive protein), hemostasis (ADAMTS, D-dimer) and immune cell types such as monocytes. Most of these biomarkers were independently associated with the air pollution levels in fully adjusted regression models. In CyTOF analyses, monocytes were enriched in participants with the highest versus the lowest PM_2.5_ exposure. In both PLS and linear regression, diastolic BP was independently associated with PM_2.5_, NO, NO_2_, CO and PAH_456_ pollution levels (*P* ≤ 0.009). Moreover, monocyte levels were independently related to both air pollution and diastolic BP levels (*P* ≤ 0.010). In in vitro cell assays, plasma of participants with high PM_2.5_ exposure induced endothelial dysfunction as evaluated by eNOS and ICAM-1 expression and tube formation.

**Conclusions:**

For the first time in adolescents, we found that ambient air pollution levels were associated with oxidative stress, acute inflammation, altered hemostasis, endothelial dysfunction, monocyte enrichment and diastolic blood pressure. Our findings provide new insights on pollution-related immunological and cardiovascular disturbances and advocate preventative measures of air pollution exposure.

## Background

The adverse health effects from exposure to ambient air pollution is a growing concern, especially given ongoing climate change [1]. Acute and chronic exposure to air pollution, particularly fine particulate matter (PM), has been shown to have a significant association with cardiovascular disease (CVD), a link that has been well-documented in adults [2]. However, there are few studies consistently showing such a linkage in children and adolescents [3, 4]. For example, PM exposure has been associated with same-day increase in systolic blood pressure (BP) in 6- to 12-year-old adolescents [5] and long-term PM exposure was found to be directly associated with increased diastolic BP in 12-year-old adolescents [6] as well as increased systolic BP and hypertension in children and adolescents aged 7 to 18 years [7]. Another study found polycylic aromatic hydrocarbons (PAH) levels associated with elevated blood pressure in adolescent boys living near a refinery in Saudi Arabia [8].

Given the prevalence of adult CVD, it is important to determine the mechanisms by which exposure to air pollution might contribute to hypertension in youth. In part, immune disturbances and oxidative stress might mediate the effects of air pollutants on the human body, including on the cardiovascular system. Previously, we demonstrated that air pollution exposure in adolescents was associated with epigenetic changes in the Foxp3 gene, a transcription factor for T regulatory cells [9, 10]. Furthermore, In a cohort of 374 Iranian children and adolescents aged 10 to 18 years, Kelishadi et al. demonstrated significant links between air quality, diet, and physical activity with markers of inflammation, oxidative stress, and insulin resistance [11]. To date, however, there are few studies describing associations between air pollution exposure, immunology and CVD in pediatric age groups. As a consequence, the underlying mechanisms by which immune disturbances might mediate the effects of air pollution on cardiovascular health remain poorly understood in adolescents and children [4].

Within a cohort of adolescents, our objectives were to: (1) identify circulating CVD and immune biomarkers of oxidative stress, such as acute inflammation; the inflammasome, the intracellular immune complex that detects external stressors and activates pro-inflammatory cytokines IL-1β, IL-18, IL-33, and IL-1F7 [12] altered hemostasis, endothelial dysfunction; and immune cell types associated with recent air pollution exposure, (2) assess the association of BP levels with both air pollution exposure and circulating CVD and immune biomarkers; (3) characterize the in vitro effects of blood plasma exposed to different levels of air pollution on endothelial cell function.

## Methods

### Study location and air pollution exposure estimation and analysis

The study area was confined to a circle with a radius of 20 km, with its center at the ambient air monitoring station located in Fresno, California, one of the most highly polluted areas in the United States, which is operated by the California Air Resources Board (CARB). Continuous daily pollutant concentrations from four air quality monitoring stations located within the Fresno city limits, periodic spatial sampling, and meteorological and geophysical data were used to assign exposures to the following pollutants as described elsewhere [13, 14]: 4-, 5-, and 6-ringed polycyclic aromatic hydrocarbons [PAH_456_], particulate matter with aerodynamic diameter of ≤2.5 μm (PM_2.5_)_,_ particulate matter with aerodynamic diameter of ≤10 μm (PM_10_), ozone (O_3_), carbon monoxide (CO), nitrogen dioxide (NO_2)_, and nitrogen oxides (NO_x_). Hourly concentrations of particle-bound PAHs were measured at each monitoring station with the PAS2000 (EcoChem Analytics, League City, TX). Spatial-temporal models that used the air quality data along with meteorological and land-use data, were used to estimate concentrations at each participant’s residence [14]. Average exposures were estimated for the week and the month preceding each participant’s clinical visit. A participant visit was scheduled on different days throughout the year. Individual exposure estimates were calculated based on the distance of the monitoring station to the participant’s home using previously published techniques [9, 15]. See Figure s1 for PM_2.5_ exposure plots. The air pollution data were subject to rigorous checks for quality assurance. These included range checks, comparison of values at nearby monitoring sites, and consistency with historical temporal and/or diurnal patterns for each pollutant.

### Study population recruitment and inclusion

During 2014, we recruited a cohort of 100 adolescents residing in Fresno, California who attended school through the Fresno Unified School District. Schools were located in the vicinity of their home geographic location increasing the yield of representative exposure data. Recruitment procedures were those previously described in the Fresno Asthmatic Children’s Environment Study (FACES) [16]. Specifically, posters and handouts were distributed throughout all 9 high schools in the Fresno District to recruit. Adolescents were also recruited through school nurses, advertisements, physicians’ offices, and local media. Inclusion criteria included participants: (1) aged 14 to 18 years; (2) residing within a 20-km radius of the CARB air quality monitoring sites in Fresno for at least 3 months; (3) who spoke English and their parents were fluent in either English or Spanish; (4) did not have plans to move from the area within the next year; and (5) resided at the provided home address at least 4 nights a week. A standardized questionnaire was used to determine their ethnicity/race, smoking behavior, and asthma status (by a parent’s report of a physician’s diagnosis). All participants gave written informed consent for the protocol that was approved by Stanford University’s Institutional Review Board.

### Study visit procedures

A single visit took place at the University of California San Francisco-Fresno, Department of Internal Medicine. During the visit, each participant was given a detailed health and demographics questionnaire, and vital signs and non-fasting blood samples were collected using validated techniques. Using the National Health and Nutrition Examination Survey protocol, BP was measured while the participant was seated and an average of 3 resting measures were taken, separated by at least 5 min. Elevated BP was defined as a BP > 120 mmHg systolic and/or > 80 mmHg diastolic, in accordance with the most recent recommendations [17].

### Blood processing

Biospecimens were shipped overnight to Stanford University and peripheral blood mononuclear cells (PBMCs) and plasma were isolated from blood samples by Ficoll density gradient centrifugation. PBMCs were stored at − 196 °C in liquid nitrogen and plasma stored at − 80 °C, per published techniques [18]. Assays included multiplex cardiovascular panels, immune panels, cell phenotyping, as well as in vitro endothelial cell plasma stimulation assays.

### Cardiovascular panels

Cardiovascular biomarkers were measured using EMD Millipore CVD panel 2 (HCVD2MAG-67 K) and 3 (HCVD3MAG-67 K). The CVD panel 2 focused on markers of oxidative stress, endothelin function, injury and hemostasis while the CVD panel 3 focused on acute-phase reactant markers. Specifically, the CVD panel 2 comprises ADAMTS13, D-Dimer, GDF-15, Myoglobin, sICAM-1, MPO, P-selectin, Lipocalin-2/NGAL, sVCAM-1, and SA.; the CVD panel 3 comprises Alpha-1 Acid Glycoprotein (AGP), Adipsin, α2-Macroglobulin, CRP, Fetuin A, Fibrinogen, L-Selectin, Serum Amyloid P, Haptoglobin, and Platelet Factor-4. The assays were performed by the Stanford Human Immune Monitoring Center (HIMC) following the manufacturer’s instructions.

### Luminex 63-Plex

The eBioscience/Affymetrix Magnetic bead kit assay was performed at Stanford HIMC using a tailored immune panel. Kits were used according to the manufacturer’s recommendations with modifications as described below. See Table s1A for the Luminex panel. Briefly, beads were added to a 96-well plate and washed in a Biotek ELx405 washer. Samples were added to the plate containing the mixed antibody-linked beads and incubated at room temperature for 1 h followed by overnight incubation at 4 °C with shaking. Cold (4 °C) and room-temperature incubation steps were performed on an orbital shaker at 500–600 rpm. Following the overnight incubation plates were washed in a Biotek ELx405 washer and then biotinylated detection antibody added for 75 min at room temperature with shaking. Plates were washed as above and streptavidin-PE was added. After incubation for 30 min at room temperature wash was performed as above and reading buffer was added to the wells. Each sample was measured in duplicate. Plates were read using a Luminex 200 instrument with a lower bound of 50 beads per sample per cytokine. Custom assay Control beads by Radix Biosolutions are added to all wells.

### High-dimensional immune and cardiovascular phenotyping

We applied CyTOF, a mass spectrometry-based method, to detect immune cell markers and deeply characterize immune cell populations and function using lanthanide-labeled specific antibodies. This technique allows staining of cells with 30–40 metal-tagged antibodies and measurement of them simultaneously within a single cell. Briefly, PBMCs were stimulated with PMA/ionomycin for 4 h, followed by surface-staining, fixation, permeabilization, and intracellular staining (e.g. for transcription factor AhR) using published techniques [19]. See Table s1B for the CyTOF panel. Samples were then acquired using a CyTOF instrument (Helios, Fluidigm). The following cellular sub-populations were identified: T regulatory (CD25 high, CD 127 low), Th1 (CCR4-, CXCR3+, CD25-), Th2 (CCR4+, CCR6-, CXCR3-, CD25-), Th17 cells (CCR4+, CCR6+, CD 161+, CXCR3-, CD25-), monocytes (CD14+), and monocytes with the aryl hydrocarbon receptor, a key biomarker in exposure to environmental toxins (CD14+, AhR+) [20]. A t-SNE-based visualization (viSNE) map was created from a maximum of 30,000 cells simultaneously. This visualization of the high-dimensional, single-cell data in a two-dimensional map provides insight into the mechanisms involved in response to pollutant exposure [21].

### In vitro endothelial cell stimulation with participants’ plasma

To determine the effects of plasma exposed to air pollution on endothelial cell function, we exposed human aortic and cardiac endothelial cells to plasma of 10 participants representing a spectrum of recent air pollution exposure levels. Human Aortic Endothelial Cell lines (HAEC) and cardiac MicroVascular Endothelial Cell lines (cMVEC) obtained from Lonza (Chicago, USA) were cultured as recommended by the manufacturer in full endothelial growth media (EGM2 and EGM2-MV) at 37 °C in a humidified atmosphere of 5% CO_2_. After 24 h of starvation with 1% of FBS, endothelial cells were incubated with or without 25% participant plasma. After 24 h of incubation, cells were collected to perform qPCR analyses or stained for in vitro angiogenesis assay. The mRNA expression of endothelial nitric oxide synthase (eNOS) and intracellular adhesion molecule 1 (ICAM-1) were measured by real-time quantitative PCR in HAEC and cMVEC to determine endothelial dysfunction. TaqMan primers for eNOS, ICAM-1, and glyceraldehyde 3-phosphate dehyrogenase (GAPDH, Applied Biosystems, Foster City, CA, USA) were used for qRT-PCR. Relative quantification was calculated by normalizing the Ct (threshold cycle) of the gene of interest to the Ct of GAPDH in the same sample, according to the comparative Ct method (∆∆Ct method). Tube formation assays were performed as described previously [22]. After thawing on ice overnight at 4 °C, 10 mg/mL Matrigel (BD) was used to coat pre-chilled 96-well plates and then incubated at 37 °C for 30 min. Each well was then seeded with HAECs and cMVEC (3 × 10^4^ cells) suspended in EGM2 containing 25% of plasma of the 10 participants. After incubation for 24 h, endothelial cells were stained with Corning Calcein AM solution at 8 μg/mL for 30 min. Microtube formation was observed and photographed under a Nikon inverted microscope. The number of tubes of each condition was normalized to the number of tubes of the untreated control cells.

### Statistical analyses

All variables were normally distributed or log-transformed to achieve normality before statistical analysis. Summary statistics were reported as mean ± SD or number (%). First, we measured the interrelation between the different air pollutants by constructing a partial correlation diagram of pollutant exposures using JMP Genomics 6.0 (SAS Institute Inc., Cary, NC). Partial regression fits covariance selection models (graphical Gaussian models) to estimate the correlation between a pair of variables adjusted for their correlation with all other variables in the network (i.e. partial correlations). As such, this method provides adjusted correlations while accounting for the complex relations of the components (pollutants) with one another. Second, using JMP Genomics v6.0, we applied partial least squares (PLS) to identify patterns of CVD biomarkers, cytokines, and immune cell types associated with pollutant exposures and BP levels. We chose this method due to its ability to deal with highly correlated predictors (e.g. cytokines). PLS creates linear combinations (latent factors) of the predictors (air pollutants, CVD biomarkers, cytokines, and immune cell types) and the outcome so that the covariance between the predictors and the outcome variables (pollutant level or elevated BP) is maximized. These latent factors are then used instead of the original individual predictors [23]. Per outcome, we selected the PLS model with the most optimal number of latent factors (which predicted the outcome best at balanced risk for under- and overfitting), as the model with the lowest predicted residuals sum of squares (PRESS) value explaining a substantial proportion of variation in both predictor and outcome variables. PRESS statistics provide a summary measure of the models’ fit and were retrieved by cross-validation, in which each observation in turn was removed and PLS models were refitted using the remaining observations. PLS is a linear regression model, but it differs from the classical multiple linear regression approach in that only the relevant part of the information present in the predictors (captured in the latent factors) is used for the prediction of outcome. The importance of each predictor (biomarker or air pollutant) in the construction of the latent factors was determined from the variable importance in projection (VIP) scores of Wold. Higher VIP score implies a higher relevance of a predictor to predict the response variable. In our analysis, predictors with a VIP > 1.4 were considered influential and were investigated in more detail. The VIP > 1.4 cut-off was based on our previous expertise using PLS for analyzing large sets of biomarkers, where a cut-off between 1.3 and 1.5 provided a good balance between number of predictors selected and number of predictors that were subsequently confirmed as predictors in multiple linear regression [24, 25] For this, we assessed in a third step the multivariable-adjusted associations of the air pollution and BP levels with the level of the biomarkers selected in PLS and compared the biomarker levels between adolescents with and without hypertension. Both the PLS and the linear regression analyses were adjusted for important confounders such as age, sex, race, body mass index (BMI), smoking status and asthma status. We accounted for asthma status given the high percentage of asthmatic children (33%) in this cohort and possible comorbidity with hypertension.

Next, we performed a mediation analysis using PROC CAUSALMED in SAS version 9.0 (Cary, NC, USA). This procedure allows estimation of potentially causal mediation effects from observational data. As such, we calculated the natural direct (NDE) and indirect effects (NIE) of the six air pollutants on diastolic BP. The NIE refers to the exposure effect (air pollutants) on the outcome variable (diastolic BP) mediated by a mediator variable (here: biomarkers linked to prior air pollution exposure in PLS). We accounted for clinical covariates (age, sex, race, BMI, smoking and asthma status) to remove confounding between the exposure, mediator and outcome variables. Here, only diastolic BP was considered as outcome as none of the air pollutants were associated with systolic BP in multiple linear regression.

For cellular and transcription factor identification, CyTOF was performed. After the CyTOF data was normalized, a t-Distributed Stochastic Neighbor Embedding (t-SNE) algorithm was run to determine immune cell clusters in an unsupervised manner using FlowSOM by R 3.6.0 programming. viSNE plots were also analyzed in the same manner [26]. Independent t-test were performed for the endothelial cellular experiments.

## Results

Table 1 shows demographic characteristics of the study population and Table 2 includes the distribution of exposure to different air pollutants. One-hundred adolescents (48% female) with a mean age of 16.1 ± 2.5 years (13.6–18.6 years) were included in the study analysis. Approximately half of the adolescents were Hispanic (53%). Mean systolic BP was 114.4 ± 14.5 mmHg and mean diastolic BP was 61.7 ± 9.6 mmHg, based on an average blood pressure calculated as the average of the two values with the smallest difference. This approach is a slight modification of the NHANES protocol [27]. A total of 34% of the adolescents had elevated BP at the time of their visit. Overall air quality was determined by the total amount of pollutants present. Table s2 shows the correlations between the measured air pollutants (PM_2.5_, PM_10_, PAH_456_, O_3_, CO, NO, and NO_2_) averaged over the 7 days before the clinic visit. While accounting for pollutant interactions, partial regression analysis showed strong and direct correlations (R) between PM_2.5_ and PM_10_ concentrations (*R* = 0.73) and between levels of CO and NO (*R* = 0.82) and NO_2_ (*R* = 0.83) (*P* < 0.0001 for all).

**Table 1:** Cohort demographics

Demographic	Value (*n* = 100)
Age (y)	16.1 ± 2.5
Female	48 (48%)
Percentile of BMI	66.6 ± 29.0
Systolic BP (mmHg)	114.4 ± 14.5
Diastolic BP (mmHg)	61.7 ± 9.6
Normotensive (BP < 120/80 mmHg)	66 (66%)
Systolic BP of Normotensive Group	105.9 ± 8.1
Diastolic BP of Normotensive Group	59.2 ± 9.0
Elevated BP (BP > 120/80 mmHg)	34 (34%)
Systolic BP of Elevated Group	130.9 ± 8.7
Diastolic BP of Elevated Group	66.5 ± 9.0
Diagnosis of asthma	33 (33%)
Ever smoked	9 (9%)
Number cigarettes smoked in lifetime	1.4 ± 0.8
Secondhand Smoke in Home	16 (16%)
*Household income*	
< $15,000	25 (25%)
$15,000 - 30,000	22 (22%)
$31,000 - 50,000	6 (6%)
> $50,000	38 (38%)
Information missing	9 (9%)
*Ethnicity/Race*	
Hispanic	53 (53%)
White Non-Hispanic	37 (37%)
African American	9 (9%)
Asian/Pacific Islander	1 (1%)

Values are mean ± SD or number (%)

**Table 2:** Distribution of exposure to pollutants

Pollutant	Median (25-75th percentile)
CO, ppm	0.29 (0.25–0.34)
NO, ppb	1.36 (1.02–2.36)
NO_2_, ppb	7.63 (6.34–9.61)
O_3_, ppb	64.1 (56.3–68.6)
PAH, ng/m^3^	2.73 (2.16–3.24)
PM_2.5_, μg/m^3^	0.96 (0.88–1.05)
PM_10_, μg/m^3^	39.3 (31.4–50.5)

*CO* carbon monoxide, *NO* nitrogen oxide, *NO*_*2*_ nitrogen dioxide, *O*_*3*_ ozone, *PAH* polycyclic aromatic hydrocarbon, *PM* particulate matter

**Fig. 1:**
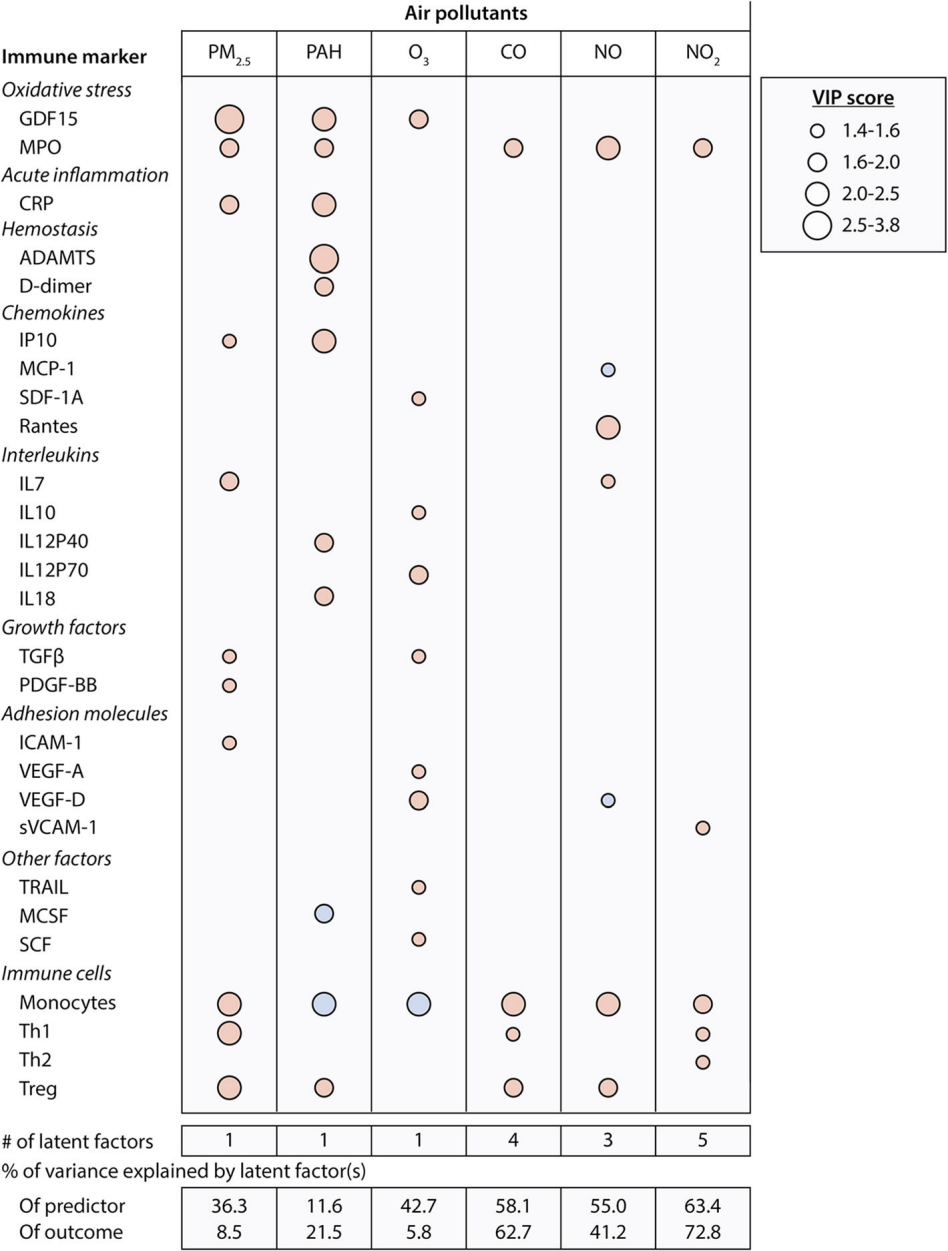
Immune markers and immune cell types associated with air pollution exposure. Heat map of immune markers and immune cells (VIP > 1.4) in PLS models explaining variability in recent exposure to air pollutants. Red are positive and blue are negative correlations. Analyses accounted for the variability in age, sex, BMI, asthma and smoking. PLS indicates partial least squares; VIP, variable importance in projection. CO, carbon monoxide; NO, nitrogen oxide; NO_2_, nitrogen dioxide; O_3_, ozone; PAH, polycyclic aromatic hydrocarbon; PM, particulate matter

**Fig. 2:**
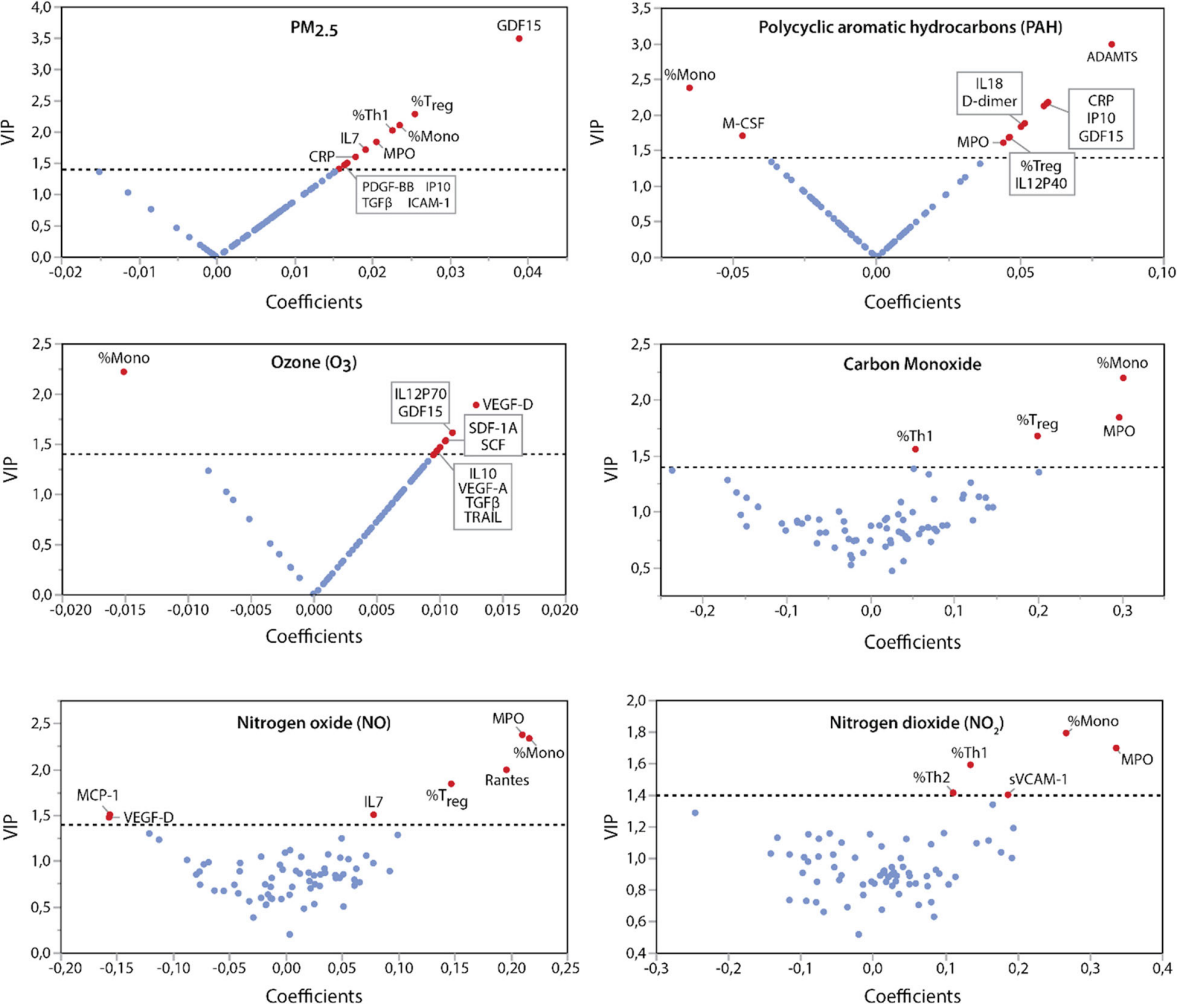
V-plots generated from continuous PLS models for air concentrations in PM_2.5_, polycyclic aromatic hydrocarbons, carbon monoxide, ozone, nitrogen oxide and nitrogen dioxide. Influential biomarkers (VIP > 1.4) are named. PLS models explained 8.5% (PM_2.5_); 21.5% (PAH), 5.8% (O_3_), 62.7% (CO), 41.2% (NO) and 72.8% (NO_2_) of the pollutants’ variance. Correlation coefficients were scaled and centered. Analyses accounted for the variability in age, sex, BMI, asthma and smoking. Mono, monocytes; Treg, T-regulatory cells; Th1, T-helper cells; CRP, C-reactive protein; MCP, Methyl-accepting chemotaxis protein; ADAMTS, A Disintegrin and Metalloproteinase with Thrombospondin motifs; MPO, myeloperoxidase; PLS, partial least squares; VIP, variable importance in projection, CRP, c-reactive protein; CO, carbon monoxide; NO, nitrogen oxide; NO_2_, nitrogen dioxide; O_3_, ozone; PAH, polycyclic aromatic hydrocarbon; PM, particulate matter

Using PLS analysis, we identified cytokines, growth factors and biomarkers of oxidative stress, acute inflammation, hemostasis, and cell types associated with air pollution levels averaged for 1 week before blood collection. PLS models explained 72.8 to 5.8% of the variability in recent air concentrations of NO_2_, CO, NO, PAH, PM_2.5_ and O_3_ in decreasing order of importance (*p* ≤ 0.025 for all comparisons; Fig. 1). Top predictors (VIP > 1.4) explaining most of the variance in recent exposure to the air pollutants included markers of oxidative stress (e.g. GDF-15 and MPO), acute inflammation (e.g. C-reactive protein, CRP), hemostasis (e.g. ADAMTS, D-dimer), inflammasome markers (e.g., IL-18) and both innate and adaptive immune regulators (e.g., monocytes and T regulatory cells). V-plots in Fig. 2 highlights the influential cytokines and immune cell types associated with each air pollutant concentration in V-plots. See Table s3 for summary data on the PLS models.

**Fig. 3:**
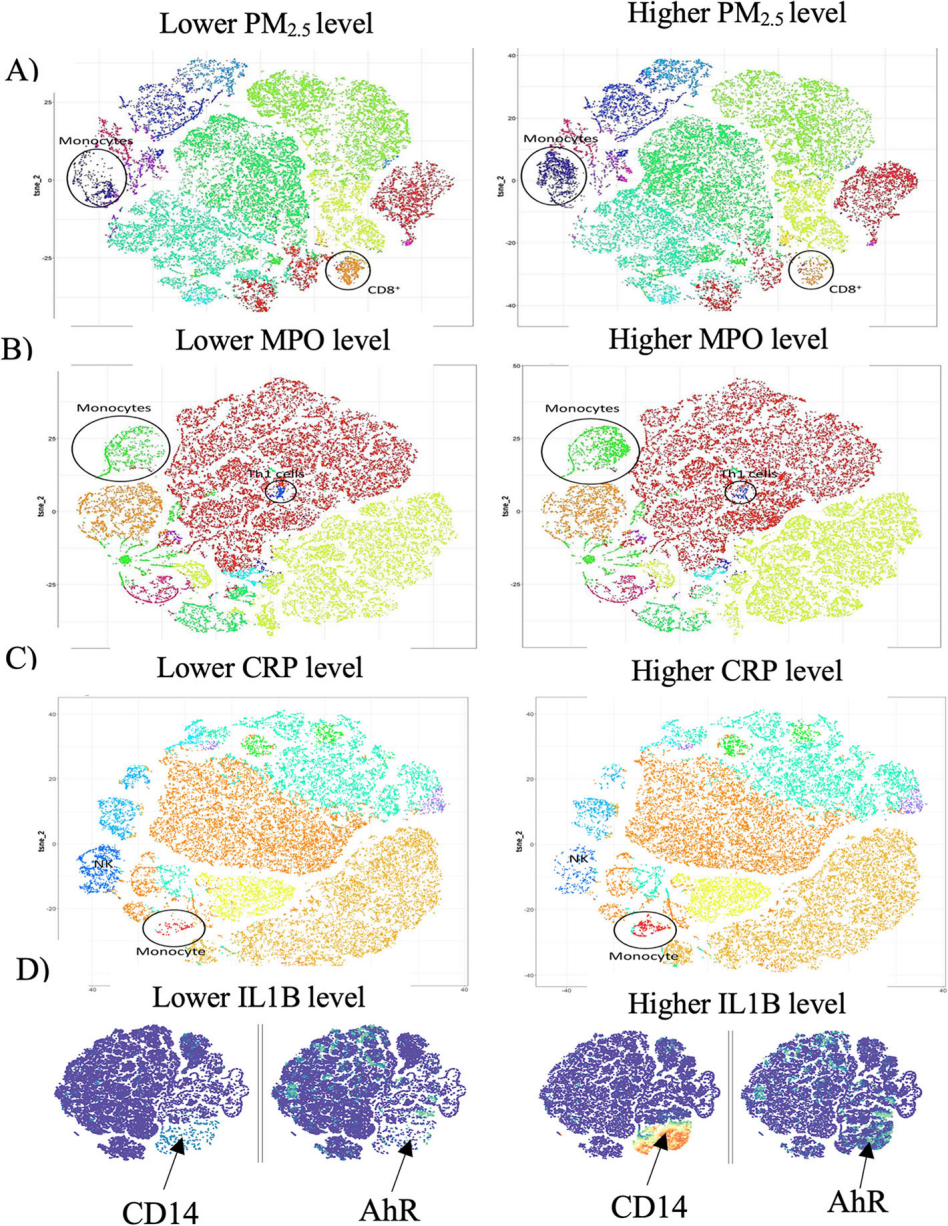
viSNE representation of CyTOF data visualizing immune cell types associated with the signatures of air pollution, oxidative stress, acute inflammation, and inflammasome activity. Increased accumulation of circulating monocyte clusters were associated with **a** high exposure to PM_2.5_, **b** elevation of myeloperoxide (MPO), **c** increased CRP, and **d** higher IL-1β levels in plasma. Panel **d** shows clustering of individual markers and visualizes an upregulation of AhR in monocytes from participants with high level of IL-1β in plasma (limited to *n* = 5 per group due to viSNE cell count maximum)

Most of the biomarkers determined in PLS analyses as influential for modelling air pollution levels were independently associated with the air pollutant levels in multivariable-adjusted analysis (Table 3). Importantly, monocyte levels were related to exposure levels of all air pollutants after full adjustment (*P* ≤ 0.033; Table 3) with positive association for PM_2.5_, CO, NO and NO_2_. With the exception of O_3_ exposure, the list of influential biomarkers and immune cell types did not differ substantially between the 1 week or 1 month average air pollution exposures (Tables s4–6). In line with the PLS analyses, in our t-SNE analysis of CyTOF data, monocytes were enriched in the participants exposed to the highest level of PM_2.5_ at 1 week prior to blood draw compared with those exposed to the lowest PM_2.5_ levels (Fig. 3a). In comparing the viSNE maps of participants with low vs. high markers of oxidative stress (MPO, Fig. 3b), acute inflammation (CRP, Fig. 3c) and the inflammasome with AhR+ enhancement (IL1-β, Fig. 3d), monocytes were significantly enriched in the groups with elevated markers [MPO (*P* = 0.01), CRP (*P* = 0.04), and IL-1β (*P* = 0.01)].

**Table 3:** Multivariable-adjusted associations between air pollution levels averaged for the week preceding the examination and their top biomarker predictors identified in Partial Least Squares analysis (PLS)

*Biomarker*	PM_2.5_ (95% CI)	PAH_456_ (95% CI)	O_3_ (95% CI)	CO (95% CI)	NO (95% CI)	NO_2_ (95% CI)
%Monocytes, per doubling	+ 0.023 (0.002 to 0.044)*	−0.15 (− 0.27 to − 0.04)*	−1.26 (− 2.31 to − 0.21)*	+ 0.029 (0.014 to 0.045)‡	+ 0.40 (0.11 to 0.69)†	+ 1.06 (0.54 to 1.58)‡
% Treg, per doubling	+ 0.085 (0.012 to 0.16)*	+ 0.26 (− 0.15 to 0.68)	− 3.21 (− 7.64 to 1.21)	+ 0.095 (0.037 to 0.15)†	+ 1.25 (0.14 to 2.36)*	+ 2.61 (0.57 to 4.64)*
% Th1, per doubling	+ 0.043 (− 0.001 to 0.085)	− 0.18 (− 0.41 to 0.06)	+ 1.33 (− 1.05 to 3.71)	0.036 (0.003 to 0.068)*	+ 0.26 (− 0.38 to 0.90)	+ 1.97 (0.87 to 3.08)‡
GDF-15, per doubling	+ 0.15 (0.07 to 0.22)‡	+ 0.35 (− 0.07 to 0.78)	+ 4.43 (0.05 to 8.81)*	+ 0.049 (− 0.012 to 0.11)	+ 0.61 (− 0.55 to 1.76)	+ 1.79 (− 0.35 to 3.93)
MPO, per doubling	+ 0.033 (0.003 to 0.064)*	+ 0.084 (− 0.080 to 0.25)	+ 0.93 (− 0.79 to 2.65)	+ 0.032 (0.010 to 0.055)†	+ 0.70 (0.28 to 1.13)†	1.45 (0.66 to 2.24)‡
CRP, per doubling	+ 0.019 (− 0.001 to 0.040)	+ 0.084 (− 0.029 to 0.20)	+ 0.97 (− 0.22 to 2.15)	− 0.006 (− 0.022 to 0.011)	−0.12 (− 0.43 to 0.19)	−0.20 (− 0.78 to 0.38)
VEGF-D, per + 66 MFI	+ 0.0034 (− 0.029 to 0.036)	+ 0.0030 (− 0.17 to 0.18)	+ 1.93 (0.17 to 3.69)*	−0.013 (0.037 to 0.011)	− 0.32 (− 0.79 to 0.14)	−0.10 (− 0.98 to 0.78)
Rantes, per + 663 MFI	+ 0.030 (− 0.29 to 0.35)	−0.053 (− 0.22 to 0.12)	−1.01 (− 2.77 to 0.75)	+ 0.021 (− 0.027 to 0.045)	+ 0.44 (− 0.018 to 0.89)	+ 0.98 (0.13 to 1.82)*
ADAMTS, per + 30 MFI	+ 0.024 (− 0.010 to 0.058)	+ 0.28 (0.10 to 0.45)†	+ 0.98 (− 0.93 to 2.90)	+ 0.0082 (− 0.018 to 0.034)	+ 0.11 (− 0.38 to 0.61)	+ 0.16 (− 0.76 to 1.09)
IL-12P70, per + 33 MFI	+ 0.014 (− 0.020 to 0.048)	+ 0.032 (− 0.16 to 0.22)	+ 2.11 (0.16 to 4.07)*	− 0.0066 (− 0.034 to 0.021)	−0.086 (− 0.58 to 0.40)	+ 0.11 (− 0.81 to 1.03)

Presented biomarkers were in the top 3 of influential markers in at least 1 of the 6 PLS models for air pollutant exposures (PM_2.5_, PAH_456_, O_3_, CO, NO and NO_2_). Values are effect sizes and their 95% CI representing the change in predicted air pollutant exposure associated with a 1-SD increase or doubling in biomarker level

*CO* carbon monoxide, *NO* nitric oxide, *O3* ozone, *PAH* polycyclic aromatic hydrocarbon, *PM* particulate matter

Markers for *P* value significance: *P < 0.05, †*P* < 0.01 and ‡ *P* < 0.001

Table 4 presents the multivariable-adjusted associations between BP and air pollutant levels averaged for 7 days prior to the study visit. After full adjustment, diastolic BP increased significantly with higher exposure to all air pollutants (*P* ≤ 0.0030) except O_3_ (*P* = 0.30). In contrast, none of the air pollutant levels were independently associated with systolic BP or with elevated BP (*P* ≥ 0.20).

**Table 4:** Multivariable-adjusted associations between Blood Pressure (BP) levels and air pollutant levels averaged in the week preceding the examination

	Systolic BP (mmHg)		Diastolic BP (mmHg)		Elevated BP	
*Air pollutant*	Effect size (95% CI)	*P* value	Effect size (95% CI)	*P* value	Odds ratio (95% CI)	*P* value
PM_2.5_, per + 0.15 μg/m^3^	− 0.52 (− 2.96 to 1.91)	0.67	2.32 (0.61 to 4.02)	0.0085^a^	0.90 (0.56 to 1.44)	0.65
PAH_456_, per + 0.80 ng/m^3^	−1.20 (−3.68 to 1.29)	0.34	2.72 (0.95 to 4.50)	0.0030^a^	1.20 (0.72 to 1.99)	0.48
O_3_, per + 8.5 ppb	0.37 (−2.21 to 2.96)	0.77	−0.51 (−2.42 to 1.40)	0.60	1.03 (0.64 to 1.67)	0.90
CO, per + 0.10 ppm	−0.83 (−3.06 to 1.39)	0.46	2.96 (1.44 to 4.49)	0.0002^a^	0.89 (0.59 to 1.34)	0.56
NO, per + 2 ppb	−0.64 (−2.91 to 1.62)	0.58	2.42 (0.85 to 3.99)	0.0030^a^	0.90 (0.59 to 1.39)	0.63
NO_2_, per + 4 ppb	−1.57 (−3.97 to 0.83)	0.20	3.87 (2.31 to 5.44)	< 0.0001^a^	1.09 (0.67 to 1.77)	0.72

Effect sizes reflect the change in BP level per 1-SD increase in air pollutant. Odds ratios for the risk of elevated BP were standardized as OR^SD^. Effect sizes and odds ratios were adjusted for age, sex, BMI, race, smoking and asthma status

^a^Remained significant after correction for multiple comparison using the Holm-Bonferroni method

**Fig. 4:**
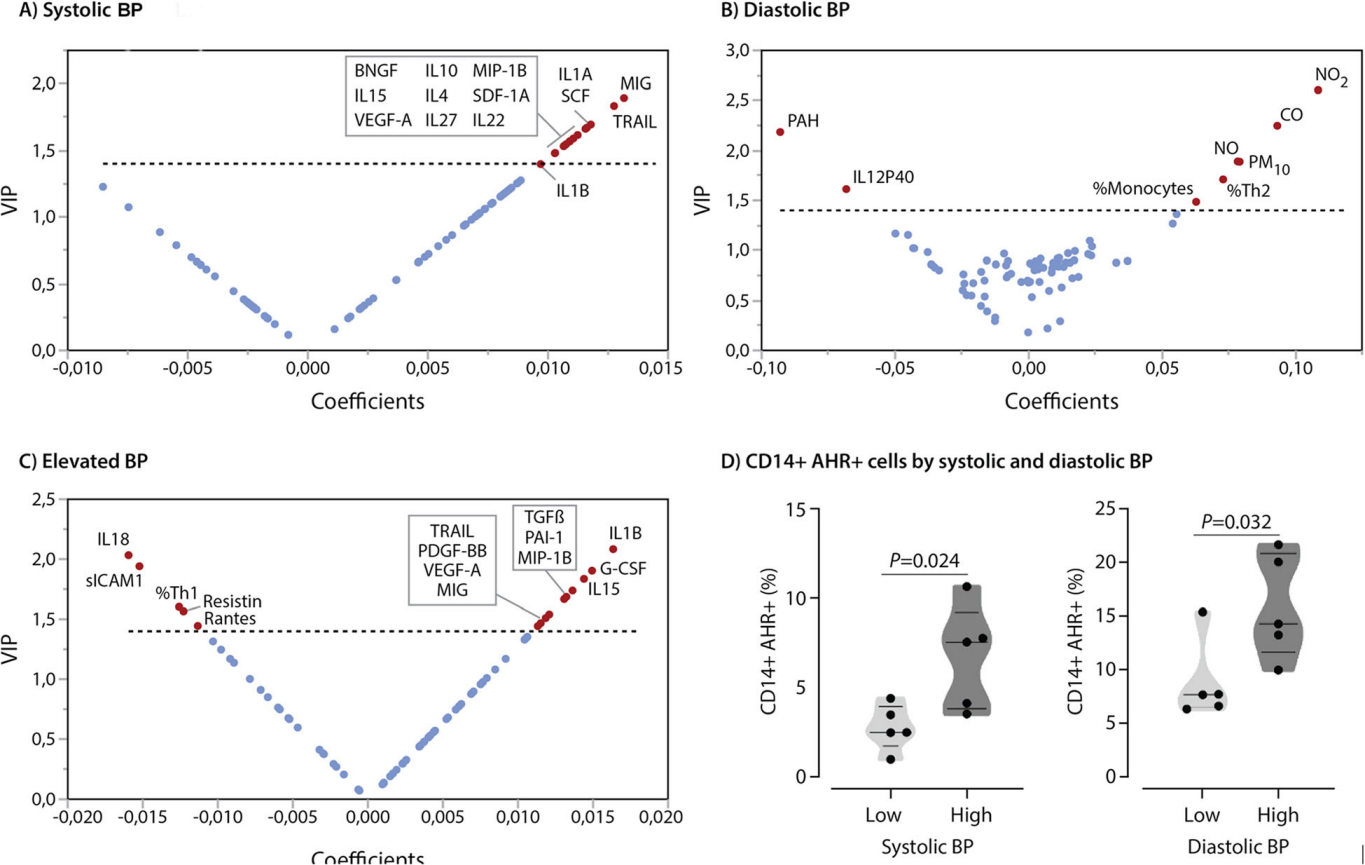
Factors associated with blood pressure (BP) levels. Panels **a**-**c**: V-plots generated from continuous PLS models for **a** systolic BP**, b** diastolic BP and **c** presence of elevated blood pressure. Influential predictors (VIP > 1.4) are named. Correlation coefficients were scaled and centered. Analyses accounted for the variability in age, sex, BMI, asthma and smoking. **d** Unpaired t-test comparing circulating AhR+ monocytes in a subset of participants (*n* = 5 per group) for both systolic BP (monocyte mean = 5.2 for low SBP vs 7.0 for high SBP) and diastolic BP (monocyte mean = 9.8 for low DBP vs 13.7 for high DBP)

Figure 4 presents the influential air pollutants, cytokines and immune cell types (VIP > 1.4) associated with BP levels as identified in PLS models, explaining 10.2% of the variance in systolic BP (Fig. 4a), 37.5% of the variance in diastolic BP (Fig. 4b), and 6.0% of the variance in elevated BP (Fig. 4c; *P* ≤ 0.026 for all). See Table s7 for summary data on the PLS models. Some of the air pollutants (NO_2_, CO, PAH_456_, NO and PM_10_) were most influential in predicting diastolic BP levels. Other top predictors for diastolic BP were IL12P40, and the percentage of Th2 cells and monocytes. Among others, the top predictor for systolic BP in PLS included CXCL9/MIG (VIP = 1.82), a cytokine produced primarily by monocytes and macrophages, and tumor necrosis factor-related apoptosis-inducing ligand (TRAIL) and IL1A. The inflammasome markers IL1-β and IL-18 were the top 2 predictors (VIP > 2) for elevated BP as a categorical variable, although predicted percentage was only 6%. Participants with elevated BP had significantly higher IL1-β average MFIs (*P* = 0.001) but not significantly higher IL-18 levels (*P* = 0.21) compared to those with normal BP. Top predictors persisted in PLS models including only cytokines and immune cells for prediction of BP levels (Table s8).

Most of the biomarkers marked as influential in PLS for modelling BP levels were also independently associated with BP levels in multivariable-adjusted analysis (Table 5). Monocyte levels were related to diastolic BP after full adjustment (*P* = 0.0019; Table 5). As such, the percentage of monocytes was related to both air pollution exposure and diastolic BP levels in multivariable-adjusted linear regression models. Other biomarkers that were influential in PLS analyses for outcome prediction were either independently related to the air pollution levels or the BP levels, but not to both. Next, we compared AHR-expressing CD14+ monocytes between 5 subjects with lowest and 5 subjects with highest levels of systolic and diastolic BP in our cohort. As shown in Fig. 4d, high level of BP was associated with a significant elevation of AhR + CD14+ circulating monocytes. These subjects were selected from children exposed to varying levels of PM2.5 to specifically investigate the potential association between blood pressure and AhR + CD14+ monocytes.

**Table 5:** Multivariable-adjusted associations between Blood Pressure (BP) Levels and their top biomarker predictors identified in Partial Least Squares analysis (PLS)

	Systolic BP (mmHg)		Diastolic BP (mmHg)	
Biomarker*	Effect size (5–95% CI)	*P* value	Effect size (5–95% CI)	*P* value
SCF, per + 40 MFI	3.30 (0.86 to 5.74)	0.0087*	0.12 (−1.75 to 1.98)	0.90
MIG, per doubling	6.06 (1.70 to 10.4)	0.0070*	1.97 (−1.35 to 5.29)	0.24
TRAIL, per doubling	7.26 (1.88 to 13.4)	0.0098*	1.88 (−2.50 to 6.25)	0.40
IL12P40, per + 271 MFI	−0.089 (− 2.59 to 2.41)	0.94	−1.57 (−3.40 to 0.22)	0.085
% Th2 cells, per doubling	0.70 (−2.22 to 0.83)	0.37	1.35 (0.35 to 2.35)	0.0086*
% monocytes, per doubling	−0.83 (−2.47 to 0.80)	0.31	1.69 (0.64 to 2.75)	0.0019*

*Presented biomarkers were within the top 3 influential markers in PLS modeling for systolic or diastolic BP levels (Table S8). Effect sizes were adjusted for age, sex, BMI, race, smoking and asthma status. *SCF* stem cell factor, *MIG* monokine induced by gamma interferon, *TRAIL* TNF-related apoptosis-inducing ligand, *MFI* median fluorescence intensity. *Remained significant after correction for multiple comparison using the Wold-Bonferroni method

**Fig. 5:**
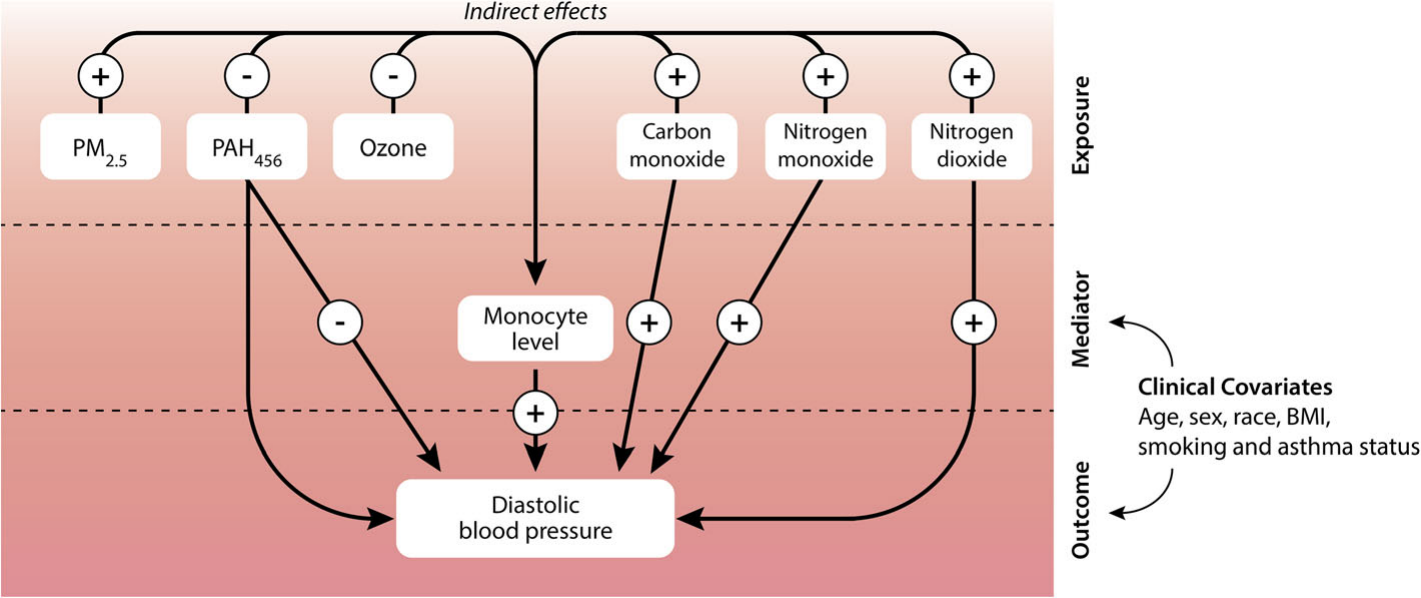
Summary of the mediation analysis with monocyte level as mediator of air pollution effects on diastolic blood pressure. Analyses accounted for variability in age, sex, race, BMI, smoking and asthma status. + and - indicate supportive and suppressive effects, respectively

Therefore, we performed a mediation analysis to examine the underlying mechanisms by which the air pollutants could influence the BP levels through a mediator, in this case through immune biomarkers. We limited our analysis to prediction of diastolic BP, as none of the air pollutants were associated with systolic BP in multiple linear regression. Furthermore, mediators included the biomarkers linked to prior air pollution exposure in PLS and multiple linear regression analyses. We accounted for clinical covariates (age, sex, race, BMI, smoking and asthma status) to remove potential confounding variables among the exposure, mediator and outcome variables. Of note, all 6 air pollutants were found to influence diastolic BP indirectly by affecting monocyte levels (Table S9, Fig. 5). Figure 5 summarizes the mediation analysis considering monocytes as mediator. In contrast, the effect of the air pollutants on diastolic BP did not seem to be mediated by the other immune markers linked to recent air pollution exposure in prior analyses (i.e. regulatory T cells, Th1 cells, GDF-15, MPO, CRP, VEGF-D, RANTES, ADAM-TS, IL-12P70), as their effects as mediator on diastolic BP were non-significant (*P* ≥ 0.093; Table S9).

To determine the effects of plasma exposed to air pollution on endothelial cell function, we exposed HAEC and cMVEC cells to plasma of 10 participants representative of different PM_2.5_ levels. There were equal number of males/females, comparable age range and ethnicity.

**Fig. 6:**
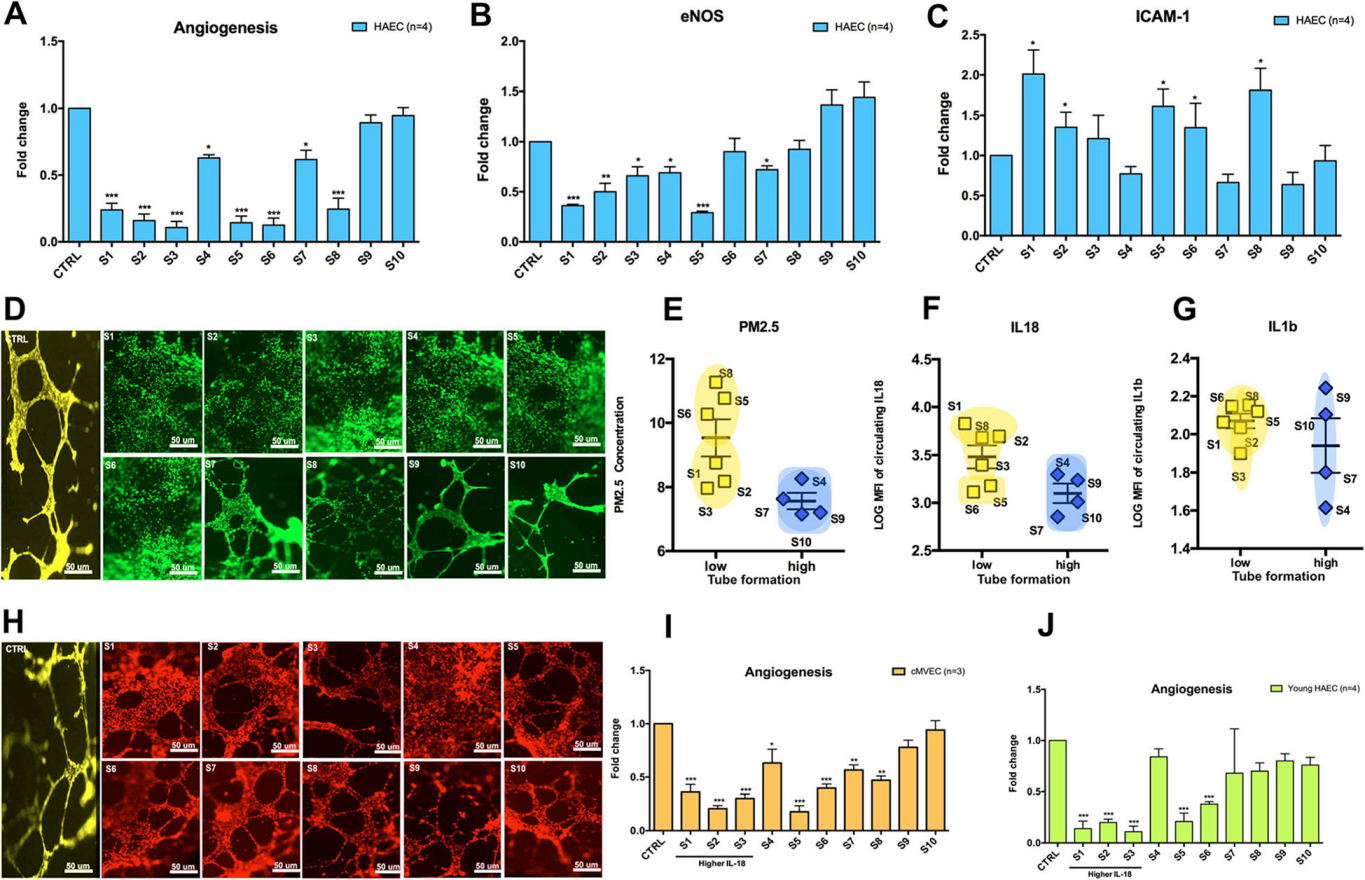
Characterization of endothelial damage from plasma of participants exposed to a spectrum of ambient PM_2.5_ levels. To characterize endothelial cell dysfunction under plasmatic stress, we starved the cells for 24 h (1% serum) and exposed them with 25% of plasma of 10 participants for 24 h in EGM2 medium. First, we evaluated microtube formation (**a**, **d**) and eNOS (**b**) and ICAM-1 (**c**) by qRT-PCR on HAEC (*n* = 4) from 50-year-old donors. Next, we evaluated if there was any association between PM_2.5_ concentration (**e**), IL-18 (**f**), or IL1-β (**g**) plasma levels from these 10 participants with angiogenesis dysfunction in HAEC. To determine if the effects showed previously with HAEC are specific, we evaluated microtube formation on cMVEC under plasma exposure (**h**, **i**)**.** To see if aging influenced endothelial cell phenotype under plasma exposure, we evaluate tube formation on HAEC of donors of 30-year-old donors (*n* = 4) (**j**)**.** Values are presented as means ± SEM. *p < 0.05; **p < 0.01, ***p < 0.001

Six plasma samples (S1, S2, S3, S5, S6, S8) induced a significant decrease in tube formation (Fig. 6a, d) and eNOS expression (Fig. 6b) and a significant increase of ICAM-1 expression (Fig. 6c) (*P* < 0.05 for all). Compared to the other 4 plasma samples, the 6 plasma samples that induced endothelial dysfunction in vitro belonged to participants that had been exposed to significantly more PM_2.5_ at 1 month prior to blood draw (*P* = 0.03; Fig. 6e). Moreover, these 6 plasma samples showed increased IL-18 expression (*P* = 0.049; Fig. 6f), but no significant difference in IL1-β expression (*P* = 0.33; Fig. 6g). These effects were independent of cell origin (Fig. 6 h, i; Figure s2) or donor age (Fig. 6 j; Figure s2).

## Discussion

In this study, we determined that ambient air pollution levels were associated with: (1) immune biomarkers reflecting pathways of oxidative stress, acute inflammation, altered hemostasis, and endothelial dysfunction; (2) monocyte enrichment, which was also independently related to diastolic BP levels; and (3) diastolic BP levels. In addition, the plasma of participants exposed to a heavily air polluted environment promoted the development of endothelial dysfunction in vitro.

Particulate matter induces oxidative stress [28, 29]. Our preliminary research using human stem-cell-derived cardiomyocytes suggests that pollutants induce significant cardiovascular toxicity by increasing the inflammatory response and oxidative stress. Here, the oxidative stress signals of growth-differentiation factor-15 (GDF-15) and myeloperoxidase (MPO) were consistent with exposure to all pollutant types we measured. GDF-15, a stress-responsive cytokine, has been shown to be a strong predictor of mortality across a wide spectrum of cardiometabolic diseases [30]. Similarly, MPO is a key biomarker associated with atherosclerotic lesions and heart failure in the general population, as highlighted by the Framingham Heart Study and other population-based adult studies [31]. This leukocyte enzyme has been implicated in cardiac remodeling and the development of heart failure in adults [32]. Such biomarkers of cardiac disease have not been previously studied in adolescents exposed to air pollution and it will be important to examine such markers in long-term longitudinal studies in children.

Particulate matter also induces inflammatory effects and immune dysregulation that may predict cardiovascular disease [33, 34]. In particular, PM_2.5_ inhalation can lead to the occurrence of systemic inflammation, increasing the risk of cardiovascular stress [35, 36]. Acute inflammation has been most often associated with circulating levels of CRP, and even predicts hypertension, suggesting hypertension is part of an inflammatory cascade [37]. PM_2.5_ is the most studied inducer of the various inflammatory pathways, such as toll-like receptor (TLR) signaling [38] that leads to systemic pro-oxidant and proinflammatory effects, which are key in the formation of atherosclerotic lesions [39]. Some of the immune markers we examined here—CRP, IL-18 IL-1β—were also statistically increased in a prior published study of adolescents exposed to wildfires in comparison to non-exposed controls [40]. In the current study, these same immune markers were among the top predictors of elevated BP in the PLS models. This finding is particularly relevant to understanding the inflammatory effects of air pollution because CRP is an independent predictor of CVD risk in adults [41], yet the association between the inflammasome and pollution and its link to CVD has not been clearly defined in adolescents.

Air pollution exposure has also shown to be associated with cardiovascular events caused by hemostasis and thrombus formation through alterations in platelet function and endothelial genes that control clot formation [42, 43]. In our study, altered hemostasis was indicated by both D-dimer and ADAMTS, suggesting that the fibrinolytic pathway involving D-dimer and a counterbalancing pro-hemostatic pathway involving ADAMTS were both activated by ambient air pollution. Studies have shown that the fibrinolytic pathway involving D-dimer may predict myocardial infarctions in adults [44, 45] and ADAMTS-5 is a extracellular matrix protease. A study by Wang, et al. found that ADAMTS was negatively associated with CVD in patients with coronary artery disease [46]. However, we found that the correlation between ADAMTS and PAH was positively correlated (+ 0.28 (0.10 to 0.45)). We suspect that differences from the Wang et al. results are related to our much younger subjects that were also free of coronary artery disease. Second, we indeed observed a positive association between ADAMTS and PAH, as well as a positive association between PAH and diastolic BP. This does not necessarily imply a positive association between ADAMTS and diastolic BP and it does not prove that ADAMTS might mediate effects of PAH on blood pressure. Based on our observations, it could be hypothesized that in adolescents ADAMTS is elevated short-term following PAH exposure as a compensatory mechanism. Regardless, how our findings in young individuals translate to elderly subjects and individuals at high risk for cardiovascular disease requires further research. Nonetheless, this is the first report showing a strong association between two key hemostasis markers and air pollution exposure in an adolescent population and is an important new finding considering that D-dimer and ADAMTS predict hypercoagulability and thrombotic events, and even moderately elevated levels of D-dimer predict long-term risk of venous and arterial events and CVD mortality [47].

We also found that air pollution exposure was associated with monocyte enrichment, consistent with a recent reported study of 13–14 year old adolescents living in a highly polluted area of China [48]. Monocytes are key white blood cells of the innate immune system and play a central role in inflammasome activation and cardiovascular disease [49, 50]. Long-term exposure to air pollution is associated with monocyte enrichment and DNA methylation in monocytes [51]. In addition, monocytes differentiate into macrophages that may accumulate in plaques and thereby play a key role in atherogenesis by promoting chronic inflammation in adults [52]. As such, monocyte enrichment may provide one type of marker for air pollution-based atherosclerosis. Moreover, the AhR pathway is activated by environmental toxic materials in a cell-specific and PM-component manner [20, 53] and AhR pathway activation was associated with higher oxidative stress, acute inflammation and IL-1β levels. In fact, the well-described downregulating effects of AhR pathway activation may be insufficient to counterbalance the pro-inflammasome effects induced by air pollution [54]. In should also be noted, however, that there were negative associations between some air pollutants and biomarkers, such as monocytes and MCSF, and further research is needed to determine the significance of these negative correlations.

In recent years, attention has focused on disease prevention, particularly risk factor modification, in younger adults. A recent U.S. analysis of 2541 teens and adults aged 15–34 found that accelerated coronary atherosclerosis was not explained by traditional risk factors in 13% of the individuals studied [55]. It is plausible that one of these yet-unidentified risk factors is exposure to air pollution, and the significant association between air pollution exposure and increased diastolic BP we observed supports this hypothesis. Hypertension has been associated with air pollution exposure in adults, and even a moderate rise in air pollutants can trigger an increase in arterial BP within a few hours [56]. Our observations suggest that air pollution affects CV health in teens, which should be taken into consideration when drafting policies on acceptable air pollution levels and determining risk factors for cardiovascular health [4].

The strong association between air pollution exposure and increased diastolic BP that we observed in adolescents may indicate the clinical expression of the cellular and mechanistic pathway changes caused by the air pollution exposure. Our mediation findings were complementary and confirmatory of our previous analyses combining feature preselection by PLS and subsequent multiple linear regression analyses. Indeed, the mediation analysis highlighted a potential key role for monocytes as mediators of air pollution effects on diastolic BP. However, our study design does not allow to infer causality and is considered a hypothesis-generating study. In addition, it is possible that pollutant exposure induces systemic inflammation and oxidative stress, which affects vascular function and, therefore, influences hemodynamic responses, ultimately leading to arterial remodeling [3, 57, 58]. It has also been shown that some constituents of PM (e.g. PM_2.5_) may pass through the alveolar capillary membrane and enter the circulatory system, directly altering the blood vessels [59]. Finally, when we stratified according to elevated BP thresholds, the IL-β pathway emerged as a relevant biomarker, although the explained percentage variability was only 6%. Our findings are consistent with the hypertensive pathophysiology literature, which identifies the activation of inflammasome pathways, endothelial dysfunction, and oxidative stress in hypertension [60]. Similar pathways are also associated with heart failure, a long-term consequence of systemic hypertension [24]. Building on our novel findings, future studies should investigate whether and to which degree the underpinnings of pathological arterial remodeling are already occurring in adolescents exposed to polluted air.

In our in vitro experiments, the plasma from adolescents exposed to high levels of air pollution induced endothelial dysfunction, possibly allowing us to classify individuals according to the spectrum of inducible endothelial toxicity. Focusing on the endothelium in air pollution-based studies is important because prior studies have consistently shown that vascular endothelial cell dysfunction, injury, and apoptosis not only play a major role in the development and progression of inflammatory vascular diseases such as atherosclerosis [61, 62], but also correlate with plaque instability and rupture, and thrombus formation [63, 64]. Previous studies have shown that air pollution exposure changes the composition of plasma enough to activate inflammatory responses, resulting in endothelial injury and angiogenesis loss. We previously demonstrated in patients with hypertension an association between left ventricular maladaptation and plasma IL-18 [24]. The endothelial-mediated injury associated with air pollution and disease in young individuals was reported in one study of 125 children and adolescents aged 10–18 years, which only used thrombomodulin and tissue factor as surrogate markers for endothelial dysfunction [65]. Plasma-induced endothelial dysfunction may be a useful surrogate marker that can provide a framework for stratifying air pollution toxicity and may serve as an indicator of cardiovascular health, much like endothelial cell counts are used as an early, specific, and independent diagnostic marker for acute coronary syndrome [66].

The Clean Air Act requires the EPA to set standards (National Ambient Air Quality Standards) for 6 air pollutants (particulate matter, ozone, sulfur oxides, carbon monoxide, nitrogen oxides and lead) based on the latest scientific research on health impacts. As such, one strength of our study was the simultaneous analysis of multiple types of these pollutants. It is noteworthy, however, that adolescents’ health was still negatively impacted despite the fact that Fresno had many days with acceptable PM_2.5_ levels based on the EPA standards (Figure s3). For the first time in an air pollution study, we employed high-dimensional mass cytometry, CyTOF [67], providing a sensitive and in-depth immunophenotyping at the single-cell level. Whereas the majority of previous air pollution-CVD studies have focused on adults, a key strength of our study was the inclusion of participants in a young age group (average age 16.1 years), allowing us to determine air pollution effects on cardiovascular and immune functioning before clinical symptomatology. Importantly, our cohorts are well-characterized using rigorous and validated measures as described previously [9, 10, 15, 68]. The cross-sectional design we employed measured prevalence of several endpoints at one timepoint, which may have been one limitation of our study. Here, this design provided an analysis of how various air pollution exposure levels and pollution types were associated with immune dysregulation and cardiovascular dysfunction. A cross-sectional analysis from a large population-based study demonstrated associations between air pollution and stroke and CVDs in adults [69]. A second limitation of our study design included a lack of validation cohort of comparable age, ethnicity, and income level in a low air pollution area, which was difficult to obtain. Importantly, it is impossible to adjust for differences of all potential variables that might influence biomarkers and blood pressure when using linear regression, such as genetics, diet, indoor air pollutants. A fourth limitation was the use of air pollution estimates and the measurement of only outdoor rather than indoor air pollution. Finally, this field of research would benefit from subsequent longitudinal studies beginning at an earlier age to understand the initial progression of inflammation.

## Conclusions

For the first time in adolescents, we found that ambient air pollution levels were associated with oxidative stress, acute inflammation, altered hemostasis, endothelial dysfunction, monocyte enrichment and diastolic BP. Importantly, we found that monocytes were also independently associated with diastolic BP, which represents a potential mechanistic link between air pollution and BP. To our knowledge, this is the first analysis that systematically characterizes differences in the patterns of immune and cardiovascular biomarkers and their associations with changes in blood pressure in a younger population exposed to ambient air pollution. Taken together, our findings suggest that ambient air pollution adversely affects CV health already early on in life, which should be taken into consideration when law makers are drafting air pollution policies.

## Supplementary information

**Additional file 1.** Supplemental Figures and Tables POLLUTION_ADOL_FINAL_Supplement.

## Data Availability

All data generated or analyzed during this study are included in this published article and its supplementary information files.
