# Correction: Immune biomarkers link air pollution exposure to blood pressure in adolescents

**DOI:** 10.1186/s12940-022-00916-1

**Published:** 2022-11-28

**Authors:** Mary Prunicki, Nicholas Cauwenberghs, Jennifer Arthur Ataam, Hesam Movassagh, Juyong Brian Kim, Tatiana Kuznetsova, Joseph C. Wu, Holden Maecker, Francois Haddad, Kari Nadeau

**Affiliations:** 1 Sean N Parker Center for Allergy and Asthma Research, Stanford University, Stanford, USA; 2 Research Unit Hypertension and Cardiovascular Epidemiology, KU Leuven Department of Cardiovascular Sciences, University of Leuven, Leuven, Belgium; 3 Research and Innovation Unit, INSERM U999, DHU TORINO, Paris Sud University, Marie Lannelongue Hospital, Le Plessis Robinson, France; 4 Institute for Immunity, Transplantation, and Infection, Stanford University, Stanford, USA; 5 Division of Cardiovascular Medicine, Department of Medicine, Stanford University, Stanford, USA; 6 Stanford Cardiovascular Institute, Stanford University School of Medicine, Stanford, USA

**Correction: Environ Health 19, 108 (2020)**

**https://doi.org/10.1186/s12940-020-00662-2**

Following the publication of the original article [1], the author reported that Figure 6 and Figure S3 should be corrected. Below are the list of modifications applied to the new Figure.

**Figure 6:**

Figure 6A: Add additional information on the Y axis title and the legend.

Figure 6B: Add additional information on the Y axis title and the legend.

Figure 6C: Add additional information on the Y axis title and the legend.

Figure 6D: Add additional information in the title of the figure and modified the

representative panels of angiogenesis.

Figure 6E: Add p value and update on the violin plot

Figure 6F: Add p value and update on the violin plot

Figure 6G: Add p value and update on the violin plot

Figure 6H: Modified the representative panels of angiogenesis.

Figure 6I: Add additional information on the Y axis title and the legend.

Figure 6J: Add additional information on the Y axis title and the legend.

**Figure S3:**

Figure S3A: Add additional information on the Y axis title and the legend.

Figure S3B: Add additional information on the Y axis title and the legend.

Figure S3C: Add additional information on the Y axis title and the legend.

Figure S3D: Add additional information on the Y axis title and the legend.

Figure S3E: Add additional information in the title of the figure and modified the

representative panels of angiogenesis.

Figure S3F: Add p value and remove the box

Figure S3G: Add p value and remove the box

Figure S3H: Add p value and remove the box

Figure S3I: Add p value and remove the box

The correct Figures are included in this correction article.

**Figure:**
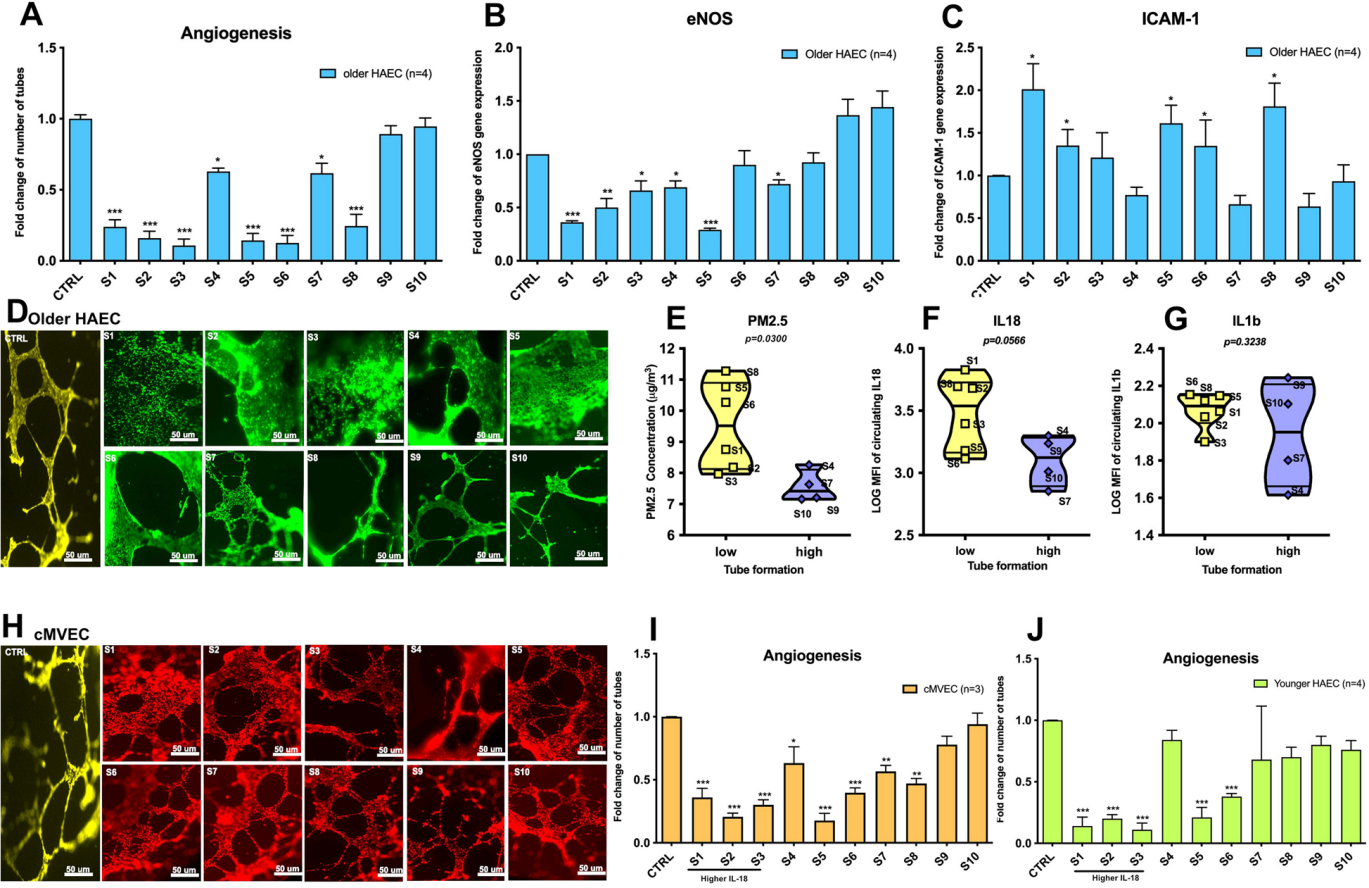
Figure 6

**Figure:**
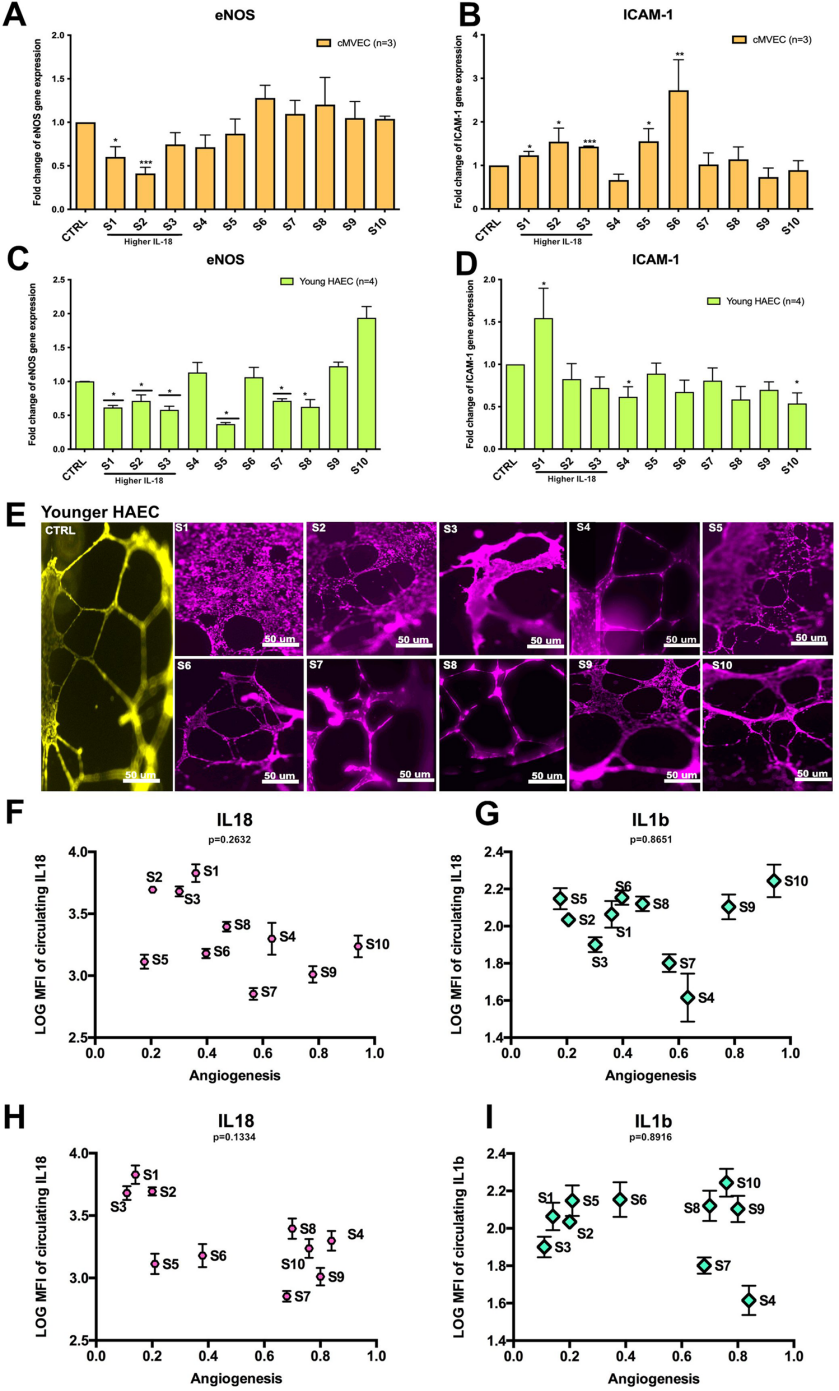
Figure S3
