# Supplementary material for: Immune biomarkers link air pollution exposure to blood pressure in adolescents
Source: Environ Health. 2020 Oct 16;19:108. doi: 10.1186/s12940-020-00662-2 (PMC7566149; doi:10.1186/s12940-020-00662-2)
Supplement: Supplementary file 1 — Additional file 1. Supplemental Figures and Tables POLLUTION_ADOL_FINAL_Supplement. [file 12940_2020_662_MOESM1_ESM.docx]

**Table s1**

A) Luminex panel

Class Cytokines

Growth factors EGF, FGF-b, HGF, NGF, PDGF-BB, PIGF-1, TGF-a, TGF-b1, VEGF-A, VEGF-D

Colony-stimulating factors G-CSF, GM-CSF, M-CSF, SCF

And stem cell factors

Interleukins IL-1a, IL-1b, IL-1RA, IL-2, IL-4, IL-5, IL-6, IL-7, IL-8, IL-9, IL-10,

IL12p40, IL12p70, IL-13,IL-15, IL-17A, IL-17F, IL-18, IL-21, IL-23, IL-27, IL-31, and LIF

Chemokines CCL2 (MCP-1), CCL3 (MIP-1a), CCL4 (MIP-1b), CCL5 (RANTES), CCL7 (MCP-3), CX/CL1 (Gro-a), CXCL5 (ENA-78), CXCL9 (MIG), CXCL10 (IP-10), CCL11 (Eotaxin), CXCL12 (SDF-1a)

Interferons INF-a, INF-b, INF-Y

Adhesion molecule ICAM-1, VCAM-1

Other factors BDNF, CD40L, FASL, leptin, PAI-1, Resistin, TNF-a, TNF-b, TRAIL

| Target Antibody | Metal | Element |
| --- | --- | --- |
| Dota-Maldemide | 115 | Ln |
| *CD49δ* | 141 | Pr |
| IL-4 | 142 | Nd |
| IL-5 | 143 | Nd |
| CD69 | 144 | Nd |
| CD4 | 145 | Nd |
| CD 8α | 146 | Nd |
| CD20 | 147 | Sm |
| CD 14 | 148 | Nd |
| *CD25* | 149 | Sm |
| Lag-3 (CD 223) | 150 | Nd |
| TCRγδ | 152 | Sm |
| CD45RA | 153 | Eu |
| CD3 | 154 | Sm |
| *CCR5* | 156 | Gd |
| *CCR4* | 158 | Gd |
| CCR7 (cd197) | 159 | Tb |
| CD 28 | 160 | Gd |
| FoxP3 | 162 | Dy |
| *CXCR3 (cd183)* | 163 | Dy |
| CD 161 | 164 | Dy |
| IFNγ | 165 | Ho |
| IL10 | 166 | Er |
| CD27 | 167 | Er |
| IL-17a | 169 | Tm |
| CD57 | 172 | Yb |
| HLA-Dr | 174 | Yb |
| PD-1 | 175 | Lu |
| CD127 (IL-7R) | 176 | Yb |
| DNA1 | 191 | Lr |
| DNA2 | 193 | Lr |

B) CyTOF panel

**Table s1. A) Luminex panel.** BDNF, brain-derived neurotrophic factor; CD40L, CD40 ligand; CCL, chemokine (C-C motif) ligand; CXCL, chemokine (C-X-C motif) ligand; EGF, epidermal growth factor; ENA-78, epithelial neutrophil-activating peptide 78; FASL, Fas ligand; FGF-b, fibroblast growth factor b; G-CSF, granulocyte colony-stimulating factor; GM-CSF, granulocyte-macrophage colony-stimulating factor; Gro-a, growth regulated a protein; HGF, hepatocyte growth factor; ICAM-1, intercellular adhesion molecule 1; IL, interleukin; IL-RA, interleukin-receptor antagonist; INF, interferon; IP-10, interferon gamma-induced protein, LIF, leukemia inhibitory factor; MCP, monocyte chemotactic protein; M-CSF, macrophage colony-stimulating factor; MIG, monokine induced by gamma interferon; MIP, macrophage inflammatory protein; NGF, nerve growth factor; PAI-1, plasminogen activator inhibitor 1; PDGF-BB, platelet-derived growth factor-BB; PIGF-1, placenta growth factor 1; RANTES, regulated on activation, normal T cell expressed and secreted; SCF, stem cell factor; SDF-1a, stromal cell-derived factor 1a; TGF, transforming growth factor; TNF, tumor necrosis factor; TRAIL, tumor necrosis factor-related apoptosis inducing ligand; VCAM-1, vascular cell adhesion molecule 1; VEGF, vascular endothelial growth factor. **B) CyTOF panel.**

**Table s2.** Partial correlations between air pollutants. CO, carbon monoxide; NO, nitrogen oxide; NO_2_, nitrogen dioxide; O_3_, ozone; PAH, polycyclic aromatic hydrocarbon; PM, particulate matter. R=correlation value.

| Correlation pair | R | *P* value |
| --- | --- | --- |
| PM_10_ and NO | 0.23 | 0.047 |
| PM_2.5_ and NO | -0.25 | 0.023 |
| PM_2.5_ and PAH | 0.16 | 0.018 |
| NO_2_ and PAH | -0.15 | 0.0018 |
| PM_2.5_ and O_3_ | 0.27 | 0.0007 |
| PM_10_ and CO | -0.43 | 0.0002 |
| O_3,_ and NO_2_ | 0.35 | <0.0001 |
| NO_2_ and NO | -0.48 | <0.0001 |
| PM_2.5_ and NO_2_ | -0.50 | <0.0001 |
| PM_2.5_ and CO | 0.53 | <0.0001 |
| PM_10_ and NO_2_ | 0.60 | <0.0001 |
| O_3_ and CO | -0.34 | <0.0001 |
| PM_10_ and PM_2.5_ | 0.73 | <0.0001 |
| CO and NO | 0.82 | <0.0001 |
| CO and NO2 | 0.83 | <0.0001 |

| Marker | **Table s3**  Air pollutant | | | | | | | | | | | | | |
| --- | --- | --- | --- | --- | --- | --- | --- | --- | --- | --- | --- | --- | --- | --- |
|  | PM_2.5_ | | PAH | | | O_3_ | | | CO | | NO | | NO_2_ | |
| *Oxidative stress* | |  | | |  | |  | | | | |  | |  |
| GDF-15 | VIP=3.50  CC=0.039 | | VIP=2.13  CC=0.058 | | | VIP=1.62  CC=0.011 | | |  | |  | |  | |
| MPO | VIP=1.84  CC=0.021 | | VIP=1.61  CC=0.044 | | |  | | | VIP=1.84  CC=0.30 | | VIP=2.38  CC=0.21 | | VIP=1.70  CC=0.34 | |
| D-dimer |  | | VIP=1.84  CC=0.050 | | |  | | |  | |  | |  | |
| ADAMTS |  | | VIP=2.99  CC=0.082 | | |  | | |  | |  | |  | |
| sVCAM-1 |  | |  | | |  | | |  | |  | | VIP=1.40  CC=0.19 | |
| *Acute inflammation* | |  | | |  | |  | | | | |  | |  |
| CRP | VIP=1.60  CC=0.018 | | VIP=2.18  CC=0.060 | | |  | | |  | |  | |  | |
| *Chemokines* | |  | | |  | |  | | | | |  | |  |
| IP10 | VIP=1.47  CC=0.016 | | VIP=2.16  CC=0.059 | | |  | | |  | |  | |  | |
| MCP-1 |  | |  | | |  | | |  | | VIP=1.51  CC=-0.16 | |  | |
| SDF-1A |  | |  | | | VIP=1.54  CC=0.011 | | |  | |  | |  | |
| Rantes |  | |  | | |  | | |  | | VIP=2.00  CC=0.20 | |  | |
| *Interleukins* |  | | |  | | | |  | |  | |  | |  |
| IL7 | VIP=1.72  CC=0.019 | |  | | |  | | |  | | VIP=1.51  CC=0.078 | |  | |
| IL10 |  | |  | | | VIP=1.47  CC=0.010 | | |  | |  | |  | |
| IL12P40 |  | | VIP=1.68  CC=0.046 | | |  | | |  | |  | |  | |
| IL12P70 |  | |  | | | VIP=1.62  CC=0.011 | | |  | |  | |  | |
| IL18 |  | | VIP=1.88  CC=0.052 | | |  | | |  | |  | |  | |
| *Growth factors* |  | |  | | |  | | |  | |  | |  | |
| TGFβ | VIP=1.50  CC=0.017 | |  | | | VIP=1.43  CC=0.010 | | |  | |  | |  | |
| PDGF-BB | VIP=1.51  CC=0.017 | |  | | |  | | |  | |  | |  | |
| *Adhesion Molecules* | |  | | |  | |  | | | | |  | |  |
| VEGF-A |  | |  | | | VIP=1.44  CC=0.010 | | |  | |  | |  | |
| VEGF-D |  | |  | | | VIP=1.89  CC=0.013 | | |  | | VIP=1.48  CC=-0.16 | |  | |
| ICAM-1 | VIP=1.41  CC=0.016 | |  | | |  | | |  | |  | |  | |
| *Other factors* |  | |  | | |  | | |  | |  | |  | |
| TRAIL |  | |  | | | VIP=1.40  CC=0.010 | | |  | |  | |  | |
| MCSF |  | | VIP=1.71  CC=-0.047 | | |  | | |  | |  | |  | |
| SCF |  | |  | | | VIP=1.53  CC=0.010 | | |  | |  | |  | |
| *Immune cells* |  | |  | | |  | | |  | |  | |  | |
| Monocytes | VIP=2.11  CC=0.024 | | VIP=2.38  CC=-0.065 | | | VIP=2.22  CC=-0.015 | | | VIP=2.20  CC=0.30 | | VIP=2.34  CC=0.22 | | VIP=1.79  CC=0.27 | |
| Th1 | VIP=2.03  CC=0.023 | |  | | |  | | | VIP=1.56  CC=0.053 | |  | | VIP=1.59  CC=0.13 | |
| Th2 |  | |  | | |  | | |  | |  | | VIP=1.42  CC=0.11 | |
| Treg | VIP=2.29  CC=0.025 | | VIP=1.69  CC=0.046 | | |  | | | VIP=1.68  CC=0.20 | | VIP=1.84  CC=0.15 | |  | |
| *N of latent factors* | 1 | | 1 | | | 1 | | | 4 | | 3 | | 5 | |
| % of variance explained by latent factors | | | | | | | | | | | |  | |  |
| Of predictors  Of outcome | 36.3  8.5 | | 11.6  21.5 | | | 42.7  5.8 | | | 58.1  62.7 | | 55.0  41.2 | | 63.4  72.8 | |
|  | | | | | | | | | | | | | | |

**Table s3.** Influential cytokines and immune cell types (VIP>1.4) retrieved from PLS analysis explaining variance in air pollutant levels measured 1 week before immune profiling. Analyses accounted for the variability in age, sex, BMI, asthma and smoking. CC indicates correlation coefficient; PLS, partial least squares; VIP, variable importance in projection. CO, carbon monoxide; NO, nitrogen oxide; NO_2_, nitrogen dioxide; O_3_, ozone; PAH, polycyclic aromatic hydrocarbon; PM, particulate matter.

**Table S4**

|  | **PM_2.5_** | | | **Polycyclic aromatic hydrocarbons (PAH)** | |
| --- | --- | --- | --- | --- | --- |
|  | **1 week before** | **1 month before** | **1 week before** | | **1 month before** |
| N of latent factors | 1 | 1 | 1 | | 1 |
| % of variation explained by latent factors: |  |  |  | |  |
| for predictor (cytokines/immune cell type) | 36.3 | 9.9 | 11.6 | | 29.3 |
| for outcome (PM_2.5_/PAH) | 8.5 | 18.3 | 21.5 | | 9.9 |
| Correlation between predicted and actual values | 0.29 | 0.42 | 0.46 | | 0.31 |
| *P* value | 0.0051 | <0.0001 | <0.0001 | | 0.0026 |
| Top predictors responsible for outcome | **GDF15** (VIP=3.50; CC=0.039)  **%Treg cells**  (VIP=2.29; CC=0.025)  **%Monocytes** (VIP=2.11; CC=0.024)  **%Th1 cells** (VIP=2.03; CC=0.023)  **MPO** (VIP=1.84; CC=0.021)  **IL7** (VIP=1.72; CC=0.019)  **CRP** (VIP=1.60; CC=0.018)  **PDGF-BB** (VIP=1.51; CC=0.017)  **TGFβ** (VIP=1.50; CC=0.017)  **IP10** (VIP=1.47; CC=0.016)  **ICAM-1** (VIP=1.41; CC=0.016) | **%Monocytes**  (VIP=2.27; CC=0.063)  **MCP-1** (VIP=2.26; CC=-0.063)  **GDF15** (VIP=2.26; CC=0.062)  **MPO** (VIP=2.22; CC=0.062)  **IL7** (VIP=2.15; CC=0.059)  **PDGF-BB** (VIP=2.13; CC=0.059)  **VEGF-D** (VIP=1.96; CC=-0.054)  **Rantes** (VIP=1.95; CC=0.054)  **EGF** (VIP=1.84; CC=0.051)  **ICAM-1** (VIP=1.67; CC=0.046)  **%Th1 cells**  (VIP=1.55; CC=0.043)  **%Treg cells**  (VIP=1.42; CC=0.039) | **ADAMTS** (VIP=2.99; CC=0.082)  **%Monocytes** (VIP=2.38; CC=-0.065)  **CRP** (VIP=2.18; CC=0.060)  **IP10** (VIP=2.16; CC=0.059)  **GDF15** (VIP=2.13; CC=0.058)  **IL18** (VIP=1.88; CC=0.052)  **D-dimer** (VIP=1.84; CC=0.050)  **M-CSF** (VIP=1.71; CC=-0.047)  **% Treg cells** (VIP=1.69; CC=0.046)  **IL12P40** (VIP=1.68; CC=0.046)  **MPO** (VIP=1.61; CC=0.044) | | **ADAMTS** (VIP=2.37; CC=0.033)  **CRP** (VIP=2.15; CC=0.030)  **IL18** (VIP=2.00; CC=0.028)  **GDF15** (VIP=1.93; CC=0.027)  **%Th1 cells** (VIP=1.83; CC=-0.026)  **%Monocytes** (VIP=1.79; CC=-0.025)  **%Treg cells** (VIP=1.78; CC=0.025)  **M-CSF** (VIP=1.73; CC=-0.024)  **IP10** (VIP=1.67; CC=0.023)  **IL12P40** (VIP=1.53; CC=0.021) |
| **Table s4:** Summary data for PLS models from cytokines and immune cell types to explain variability in air pollution by PM_2.5_ and polycyclic aromatic hydrocarbons measured either 1 week and 1 month before the immune profiling. Analyses accounted for the variability in age, sex, BMI, asthma and smoking. CC indicates correlation coefficient; PLS, partial least squares; VIP, variable importance in projection. | | | | | |

**Table s5**

|  | **Ozone (O_3_)** | | **Carbon monoxide (CO)** | |
| --- | --- | --- | --- | --- |
|  | **1 week before** | **1 month before** | **1 week before** | **1 month before** |
| N of latent factors | 1 | 1 | 4 | 3 |
| % of variation explained by latent factors |  |  |  |  |
| for predictor (cytokines/immune cell type) | 42.7 | 37.0 | 58.0 | 51.9 |
| for outcome (O_3_/CO) | 5.8 | 7.9 | 62.7 | 52.7 |
| Correlation between predicted and actual values | 0.24 | 0.27 | 0.79 | 0.72 |
| *P* value | 0.025 | 0.010 | <0.0001 | <0.0001 |
| Top predictors responsible for outcome | **%Monocytes** (VIP=2.22; CC=-0.015)  **VEGF-D** (VIP=1.89; CC=0.013)  **IL12P70** (VIP=1.62; CC=0.011)  **GDF15**  (VIP=1.62; CC=0.011)  **SDF-1A** (VIP=1.54; CC=0.011)  **SCF** (VIP=1.53; CC=0.010)  **IL10** (VIP=1.47; CC=0.010)  **VEGF-A** (VIP=1.44; CC=0.010)  **TGFβ** (VIP=1.43; CC=0.010)  **TRAIL** (VIP=1.40; CC=0.010) | **IFNγ** (VIP=2.82; CC=0.028)  **IL18** (VIP=2.66; CC=0.026)  **MPO**  (VIP=2.60; CC=0.025)  **IL1RA** (VIP=2.24; CC=0.022)  **%Monocytes** (VIP=1.64; CC=-0.016)  **Eotaxin** (VIP=1.61; CC=0.016)  **IL17A** (VIP=1.47; CC=0.014)  **PAI-1** (VIP=1.42; CC=0.014) | **%Monocytes** (VIP=2.20; CC=0.30)  **MPO** (VIP=1.85; CC=0.30)  **%Treg cells** (VIP=1.68; CC=-0.20)  **%Th1 cells** (VIP=1.56; CC=0.053) | **%Monocytes** (VIP=3.52; CC=0.35)  **%Treg cells** (VIP=2.11; CC=0.17)  **ICAM-1** (VIP=1.96; CC=0.12)  **Rantes** (VIP=1.89; CC=0.17)  **IL18** (VIP=1.58; CC=-0.16)  **%Th1 cells** (VIP=1.46; CC=0.038) |
| **Table s5.** Summary data for PLS models from cytokines and immune cell types to explain variability in air pollution by ozone and carbon monoxide measured either 1 week and 1 month before the immune profiling. Analyses accounted for the variability in age, sex, BMI, asthma and smoking. CC indicates correlation coefficient; PLS, partial least squares; VIP, variable importance in projection. | | | | |

**Table s6**

|  | **Nitrogen monoxide (NO)** | | **Nitrogen dioxide (NO_2_)** | |
| --- | --- | --- | --- | --- |
|  | **1 week before** | **1 month before** | **1 week before** | **1 month before** |
| N of latent factors | 3 | 3 | 5 | 4 |
| % of variation explained by latent factors |  |  |  |  |
| for predictor (cytokines/immune cell type) | 55.0 | 51.3 | 63.4 | 58.0 |
| for outcome (O_3_/CO) | 41.2 | 50.5 | 72.8 | 66.4 |
| Correlation between predicted and actual values | 0.64 | 0.70 | 0.85 | 0.82 |
| *P* value | <0.0001 | <0.0001 | <0.0001 | <0.0001 |
| Top predictors responsible for outcome | **MPO** (VIP=2.38; CC=0.21)  **%Monocytes** (VIP=2.34; CC=0.22)  **Rantes**  (VIP=2.00; CC=0.20)  **%Treg cells** (VIP=1.84; CC=0.15)  **MCP-1** (VIP=1.51; CC=-0.16)  **IL7** (VIP=1.51; CC=0.078)  **VEGF-D** (VIP=1.48; CC=-0.16) | **%Monocytes** (VIP=3.44; CC=0.33)  **%Treg cells** (VIP=2.16; CC=0.16)  **Rantes**  (VIP=2.15; CC=0.20)  **MPO** (VIP=1.64; CC=0.20)  **VEGF-D** (VIP=1.57; CC=-0.17)  **%Th1 cells** (VIP=1.45;  CC=-0.002)  **ENA78** (VIP=1.42; CC=-0.14) | **%Monocytes** (VIP=1.79; CC=0.27)  **MPO** (VIP=1.70; CC=0.34)  **% Th1 cells** (VIP=1.59; CC=0.13)  **% Th2 cells** (VIP=1.42; CC=0.11)  **sVCAM-1** (VIP=1.40; CC=0.19) | **%Monocytes** (VIP=2.28; CC=0.38)  **MPO** (VIP=1.66; CC=0.30)  **%Th2 cells** (VIP=1.40; CC=0.074) |
| **Table s6.** Summary data for PLS models from cytokines and immune cell types to explain variability in air pollution by nitrogen oxides measured either 1 week and 1 month before the immune profiling. Analyses accounted for the variability in age, sex, BMI, asthma and smoking. CC indicates correlation coefficient; PLS, partial least squares; VIP, variable importance in projection. | | | | |

**Table s7**

|  | **Systolic BP** | | **Diastolic BP** | **Hypertension** |
| --- | --- | --- | --- | --- |
| N of latent factors | 1 | 2 | | 1 |
| % of variation explained by latent factors |  |  | |  |
| for predictor (cytokines/immune cells/pollutants) | 40.4 | 46.4 | | 36.7 |
| for outcome (BP) | 10.2 | 37.5 | | 6.0 |
| Correlation between predicted and actual values | 0.29 | 0.52 | | 0.22 |
| *P* value | 0.0040 | <0.0001 | | 0.026 |
| Top predictors responsible for outcome | **MIG** (VIP=1.82; CC=0.014)  **TRAIL** (VIP=1.71; CC=0.013)  **IL1A** (VIP=1.67; CC=0.012)  **SCF** (VIP=1.59; CC=0.012)  **BNGF** (VIP=1.53; CC=0.011)  **IL15** (VIP=1.52; CC=-0.011)  **VEGF-A** (VIP=1.51; CC=0.011)  **IL10** (VIP=1.50; CC=0.011)  **IL4** (VIP=1.49; CC=0.011)  **IL27** (VIP=1.49; CC=0.011)  **MIP-1B** (VIP=1.43; CC=-0.011)  **SDF-1A** (VIP=1.49; CC=0.011)  **IL22** (VIP=1.43; CC=0.011)  **IL1B**  (VIP=1.40; CC=0.010) | **NO_2_** (VIP=2.60; CC=0.11)  **CO** (VIP=2.25; CC=0.093)  **PAH** (VIP=2.19; CC=-0.093)  **NO** (VIP=1.89; CC=0.078)  **PM10** (VIP=1.89; CC=0.079)  **%Th2 cells** (VIP=1.71; CC=0.073)  **IL12P40** (VIP=1.61; CC=-0.068)  **%Monocytes**  (VIP=1.49, CC= 0.063) | | **IL1B** (VIP=2.09; CC=0.016)  **IL18** (VIP=2.04; CC=-0.016)  **sICAM1** (VIP=1.95; CC=-0.015)  **G-CSF** (VIP=1.91; CC=0.015)  **IL15** (VIP=1.84; CC=0.014)  **TGFβ** (VIP=1.74; CC=0.013)  **PAI-1** (VIP=1.69; CC=0.013)  **MIP-1B** (VIP=1.67; CC=0.013)  **%Th1 cells** (VIP=1.61; CC=-0.012)  **Resistin** (VIP=1.57; CC=-0.012)  **TRAIL** (VIP=1.55; CC=0.012)  **PDGF-BB** (VIP=1.52; CC=0.012)  **VEGF-A** (VIP=1.47; CC=0.011)  **Rantes** (VIP=1.45; CC=-0.011)  **MIG** (VIP=1.45; CC=0.011) |
| **Table s7.** Summary data for PLS models from air pollutants, cytokines and immune cell types to predict blood pressure (BP) levels and hypertensive status. Analyses accounted for the variability in age, sex, BMI, asthma and smoking. CC indicates correlation coefficient; PLS, partial least squares; VIP, variable importance in projection. | | | | |

**Table s8**

|  | **Systolic BP** | **Diastolic BP** | **Hypertension** |
| --- | --- | --- | --- |
| N of latent factors | 1 | 1 | 1 |
| % of variation explained by latent factors |  |  |  |
| for predictor (cytokines/immune cells) | 44.3 | 37.8 | 42.5 |
| for outcome (BP) | 7.1 | 8.3 | 3.4 |
| Correlation between predicted and actual values | 0.26 | 0.27 | 0.18 |
| *P* value | 0.0088 | 0.0084 | 0.073 |
| Top predictors responsible for outcome | **MIG** (VIP=1.78; CC=0.010)  **TRAIL** (VIP=1.79; CC=0.010)  **SCF** (VIP=1.64; CC=0.010)  **IL1A** (VIP=1.62; CC=0.010)  **BNGF** (VIP=1.62; CC=0.010)  **IL4** (VIP=1.56; CC=0.009)  **IL15** (VIP=1.54; CC=0.009)  **IL27** (VIP=1.50; CC=0.009)  **IL10** (VIP=1.49; CC=0.009)  **IL1B** (VIP=1.47; CC=0.009)  **MIP-1B** (VIP=1.46; CC=0.009)  **VEGF-A** (VIP=1.45; CC=0.009) | **%Monocytes** (VIP=2.72; CC=0.027)  **%Th2 cells** (VIP=2.19; CC=0.022)  **IL12-P40** (VIP=1.77; CC=-0.018)  **%Th17 cells** (VIP=1.69; CC=0.017)  **GROA** (VIP=1.66; CC=0.017)  **IL5** (VIP=1.64; CC=0.016)  **IL18** (VIP=1.57; CC=-0.016)  **sFas** (VIP=1.52; CC=0.015)  **PDGF-BB** (VIP=1.46; CC=0.015)  **%Th1 cells** (VIP=1.42; CC=0.014) | **PAI-1** (VIP=2.07; CC=0.011)  **IL1B** (VIP=1.98; CC=0.011)  **TGFβ** (VIP=1.87; CC=0.010)  **SCF** (VIP=1.85; CC=0.010)  **IL15** (VIP=1.84; CC=0.010)  **G-CSF** (VIP=1.81; CC=0.010)  **MIP-1B** (VIP=1.67; CC=0.009)  **ADAMTS** (VIP=1.62; CC=-0.009)  **TNFα** (VIP=1.49; CC=0.008)  **TRAIL** (VIP=1.44; CC=0.008)  **MIG** (VIP=1.43; CC=0.008)  **IL1A** (VIP=1.43; CC=0.008) |
| **Table s8.** Summary data for PLS models from cytokines and immune cells to predict blood pressure blood pressure (BP) levels and hypertensive status. Analyses accounted for the variability in age, sex, BMI, asthma and smoking. CC indicates correlation coefficient; PLS, partial least squares; VIP, variable importance in projection. | | | |

**Table S9. Mediation analysis to identify direct and indirect effects of air pollution exposure on diastolic blood pressure (BP).** Immune markers linked to recent air pollution exposure (see Figure 1, Table 2) were considered as potential mediators.

| Exposure 🡺 Mediator | NDE | NIE | % mediated | Mediator model | | | Outcome model (diastolic BP) | | |
| --- | --- | --- | --- | --- | --- | --- | --- | --- | --- |
|  |  |  |  | Effect exposure | *P* value | Effect  exposure | *P* value | Effect mediator | *P* value |
| PM_2.5_ 🡺 monocytes | 9.23% | 3.16% | 25.5% | 0.11±0.05 | 0.027 | 1.48±0.84 | 0.078 | 2.13±0.80 | 0.0078 |
| PAH_456_ 🡺 monocytes | -2.71%* | 0.56% | 17.1% | -0.12±0.05 | 0.0064 | -2.25±0.82 | 0.0063 | 1.70±0.85 | 0.045 |
| O_3_ 🡺 monocytes | -0.04% | -0.07% | 61.8% | -0.12±0.05 | 0.015 | -0.36±0.85 | 0.68 | 2.25±0.84 | 0.0078 |
| CO 🡺 monocytes | 24.1%* | 4.2% | 14.7% | 0.18±0.05 | <0.0001 | 2.89±0.86 | 0.0008 | 1.24±0.84 | 0.14 |
| NO 🡺 monocytes | 0.91%* | 0.23% | 20.5% | 0.13±0.05 | 0.0052 | 2.07±0.79 | 0.010 | 1.87±0.80 | 0.019 |
| NO_2_ 🡺 monocytes | 0.83%* | 0.10% | 10.9% | 0.19±0.05 | <0.0001 | 3.60±0.82 | <0.0001 | 1.07±0.78 | 0.17 |
| PM_2.5_ 🡺 regulatory T cells | 12.4%* | -0.01% | 0.12% | 0.052±0.025 | 0.017 | 1.76±0.86 | 0.023 | -0.01±0.83 | 0.99 |
| PAH_456_ 🡺 regulatory T cells | -3.42%* | 0.17% | 5.06% | 0.022±0.014 | 0.11 | -2.85±0.81 | 0.0005 | 0.81±0.80 | 0.32 |
| O_3_ 🡺 regulatory T cells | -0.11% | -0.01% | 4.36% | -0.014±0.014 | 0.29 | -0.94±0.85 | 0.30 | 0.36±0.88 | 0.68 |
| CO 🡺 regulatory T cells | 29.6%* | -1.33% | 4.72% | 0.037±0.013 | 0.0052 | 3.55±0.84 | <0.0001 | -0.55±0.83 | 0.51 |
| NO 🡺 regulatory T cells | 1.16%* | -0.01% | 0.91% | 0.030±0.014 | 0.036 | 2.63±0.82 | 0.0012 | -0.11±0.81 | 0.89 |
| NO_2_🡺 regulatory T cells | 0.96%* | -0.029% | 3.06% | 0.032±0.014 | 0.020 | 4.17±0.78 | <0.0001 | -0.50±0.74 | 0.50 |
| PM_2.5_ 🡺 T helper cells 1 | 11.0%* | 1.37% | 11.1% | 0.050±0.012 | 0.036 | 1.76±0.86 | 0.040 | 0.98±0.82 | 0.24 |
| PAH_456_ 🡺 T helper cells 1 | -3.09%* | -0.17% | 5.18% | -0.034±0.025 | 0.17 | -2.56 ±0.81 | 0.0015 | 0.96±0.80 | 0.23 |
| O_3_ 🡺 T helper cells 1 | -0.13% | 0.023% | 20.6% | 0.032±0.026 | 0.20 | -1.13±0.85 | 0.19 | 1.40±0.84 | 0.096 |
| CO 🡺 T helper cells 1 | 27.3%* | 1.05% | 3.71% | 0.053±0.025 | 0.036 | 3.28±0.82 | <0.0001 | 0.55±0.79 | 0.49 |
| NO 🡺 T helper cells 1 | 1.11%* | 0.039% | 3.40% | 0.018±0.025 | 0.47 | 2.52±0.79 | 0.0014 | 1.15±0.78 | 0.14 |
| NO_2_🡺 T helper cells 1 | 0.93%* | 0.001% | 0.12% | 0.082±0.23 | 0.0006 | 4.04±0.78 | <0.0001 | 0.014±0.77 | 0.99 |
| PM_2.5_ 🡺 GDF-15 | 17.1%* | -1.70% | 11.0% | 0.050±0.012 | <0.0001 | 2.74±0.96 | 0.0041 | -0.66±0.90 | 0.47 |
| PAH_456_ 🡺 GDF-15 | -3.44% | 0.17% | 5.36% | 0.024±0.012 | 0.059 | -2.86±0.89 | 0.0014 | 0.73±0.86 | 0.40 |
| O_3_ 🡺 GDF-15 | -0.070 | 0.010 | 17.3% | 0.028±0.013 | 0.028 | -0.60±0.94 | 0.52 | 0.38±0.91 | 0.68 |
| CO 🡺 GDF-15 | 29.0%* | -0.33% | 1.17% | 0.019±0.013 | 0.15 | 3.48±0.88 | <0.0001 | -0.25±0.83 | 0.76 |
| NO 🡺 GDF-15 | 1.19%* | 0.005% | 0.38% | 0.012±0.013 | 0.34 | 2.70±0.86 | 0.0018 | 0.10±0.83 | 0.90 |
| NO_2_🡺 GDF-15 | 0.95%* | -0.009% | 0.99% | 0.020±0.013 | 0.13 | 4.12±0.82 | <0.0001 | -0.25±0.78 | 0.75 |
| PM_2.5_ 🡺 MPO | 14.6%* | 0.85% | 5.51% | 0.075±0.034 | 0.023 | 2.34±0.90 | 0.0098 | 0.60±0.90 | 0.51 |
| PAH_456_ 🡺 MPO | -3.51* | 0.24% | 7.42% | 0.045±0.034 | 0.19 | -2.91±0.87 | 0.0008 | 1.48±0.88 | 0.093 |
| O_3_ 🡺 MPO | -0.080% | 0.020% | 33.9% | 0.046±0.034 | 0.17 | -0.68±0.94 | 0.46 | 1.24±0.93 | 0.18 |
| CO 🡺 MPO | 28.0%* | 0.64% | 2.23% | 0.084±0.033 | 0.014 | 3.36±0.90 | 0.0002 | 0.30±0.89 | 0.73 |
| NO 🡺 MPO | 1.15%* | 0.043% | 3.57% | 0.10±0.032 | 0.0018 | 2.61±0.91 | 0.0039 | 0.32±0.92 | 0.73 |
| NO_2_🡺 MPO | 0.96%* | -0.017 | 1.85% | 0.11±0.032 | 0.0008 | 4.15±0.86 | <0.0001 | -0.23±86 | 0.79 |
| PM_2.5_ 🡺 C-reactive protein | 16.9%* | -1.48% | 9.61% | 0.093±0.050 | 0.062 | 2.70±0.89 | 0.0023 | -1.50±1.06 | 0.16 |
| PAH_456_ 🡺 C-reactive protein | -3.19%* | -0.079% | 2.42% | 0.098±0.050 | 0.051 | -2.65±0.90 | 0.0031 | -0.39±1.09 | 0.71 |
| O_3_ 🡺 C-reactive protein | -0.037% | -0.023% | 38.8% | 0.094±0.049 | 0.047 | -0.32±0.93 | 0.74 | -1.20±1.16 | 0.30 |
| CO 🡺 C-reactive protein | 28.0%* | 0.66% | 2.30% | -0.060±0.051 | 0.24 | 3.36±0.87 | 0.0001 | -0.78±1.06 | 0.46 |
| NO 🡺 C-reactive protein | 1.17%* | 0.021% | 1.79% | -0.052±0.050 | 0.29 | 2.66±0.86 | 0.0021 | -0.54±1.05 | 0.60 |
| NO_2_🡺 C-reactive protein | 0.93%* | 0.0075% | 0.79% | -0.056±0.052 | 0.27 | 4.04 ±0.82 | <0.0001 | -0.34±0.99 | 0.73 |
| PM_2.5_ 🡺 VEGF-D | 15.5%* | -0.083% | 0.54% | 1.47±7.07 | 0.84 | 2.48±0.88 | 0.0047 | 0.17±0.86 | 0.84 |
| PAH_456_ 🡺 VEGF-D | -3.26%* | -0.001% | 0.037 | 0.10±7.12 | 0.99 | -2.71±0.87 | 0.0020 | -0.64±0.85 | 0.45 |
| O_3_ 🡺 VEGF-D | -0.043% | -0.017% | 28.2% | 15.3±6.9 | 0.028 | -0.37±0.94 | 0.70 | -0.62±0.92 | 0.50 |
| CO 🡺 VEGF-D | 28.3%* | 0.33% | 1.17% | -7.43±7.22 | 0.30 | 3.40±0.87 | 0.0001 | -0.35±0.85 | 0.67 |
| NO 🡺 VEGF-D | 1.18%* | 0.011% | 0.94% | -9.72±6.90 | 0.16 | 2.68±0.86 | 0.0021 | -0.17±0.85 | 0.84 |
| NO_2_🡺 VEGF-D | 0.94%* | 0.0025% | 0.26% | -1.55±7.07 | 0.83 | 4.09±0.82 | <0.0001 | -0.46±0.79 | 0.56 |
| PM_2.5_ 🡺 RANTES | 15.4%* | 0.069% | 0.45% | 12.3±72.0 | 0.86 | 12.3±72.0 | 0.0052 | 0.60±0.86 | 0.48 |
| PAH_456_ 🡺 RANTES | -3.22%* | -0.043% | 1.32% | -51.0±72.3 | 0.48 | -2.67±0.88 | 0.0023 | 0.46±0.86 | 0.58 |
| O_3_ 🡺 RANTES | -0.10% | -0.017% | 14.4% | -103.1±71.6 | 0.15 | -0.85±1.02 | 0.39 | 0.93±0.99 | 0.33 |
| CO 🡺 RANTES | 28.6%* | -0.001% | 0.002% | 138.3±73.0 | 0.058 | 3.43±0.89 | 0.0001 | -0.27±0.86 | 0.99 |
| NO 🡺 RANTES | 1.18* | 0.0098% | 0.83% | 141.2±69.5 | 0.042 | 2.68±0.89 | 0.0023 | 0.11±0.86 | 0.90 |
| NO_2_🡺 RANTES | 0.96%* | -0.020% | 2.16% | 171.4±69.9 | 0.014 | 4.17±0.82 | <0.0001 | -0.33 ±0.80 | 0.67 |
| PM_2.5_ 🡺 ADAM-TS | 16.4%* | -0.92 | 5.93% | 4.38±3.02 | 0.15 | 2.62±0.88 | 0.0031 | -0.99±0.90 | 0.26 |
| PAH_456_ 🡺 ADAM-TS | -3.40%* | 0.13% | 4.10% | 9.63±2.89 | 0.0009 | -2.82±0.93 | 0.0024 | 0.36±0.97 | 0.72 |
| O_3_ 🡺 ADAM-TS | -0.055% | -0.005% | 8.09% | 3.48±2.98 | 0.25 | -0.47±0.94 | 0.61 | -0.36±0.96 | 0.71 |
| CO 🡺 ADAM-TS | 28.9%* | -0.26% | 0.92% | 1.57±3.11 | 0.61 | 3.46±0.87 | <0.0001 | -0.60±0.87 | 0.49 |
| NO 🡺 ADAM-TS | 1.20%* | -0.013% | 1.11% | 1.25±3.00 | 0.68 | 2.72±0.86 | 0.0014 | -0.72±0.87 | 0.41 |
| NO_2_🡺 ADAM-TS | 0.94%* | -0.005% | 0.51% | 0.85±3.05 | 0.78 | 4.10±0.81 | <0.0001 | -0.72±0.81 | 0.38 |
| PM_2.5_ 🡺 IL-12P70 | 15.4%* | 0.25% | 1.61% | 2.93±3.38 | 0.39 | 2.43±0.88 | 0.0060 | 0.046±0.88 | 0.61 |
| PAH_456_ 🡺 IL-12P70 | -3.31%* | 0.042% | 1.30% | 1.28±3.28 | 0.70 | -2.75±0.87 | 0.0017 | 0.92±0.92 | 0.32 |
| O_3_ 🡺 IL-12P70 | -0.088% | 0.029% | 46.7% | 7.16±3.07 | 0.021 | -0.75±0.94 | 0.42 | 1.09±1.02 | 0.28 |
| CO 🡺 IL-12P70 | 29.2%* | -0.56% | 1.94% | -1.93±3.26 | 0.55 | 3.50±0.86 | <0.0001 | 1.15±0.92 | 0.21 |
| NO 🡺 IL-12P70 | 1.21%* | -0.015% | 1.28% | -1.45±3.34 | 0.66 | 2.75±0.86 | 0.0014 | 0.79±0.86 | 0.37 |
| NO_2_🡺 IL-12P70 | 0.94% | 0.0019% | 0.20% | 0.43±3.39 | 0.90 | 4.08±0.82 | <0.0001 | 0.63±0.82 | 0.45 |

Effect sizes ± standard error reflect the effect of a 1-SD increase in exposure/mediator on the mediator/outcome. We accounted for clinical covariates (age, sex, race, BMI, smoking and asthma status) to remove confounding between the exposure, mediator and outcome variables. NDE, natural direct effect; NIE, natural indirect effect.

**Figure s1**

**
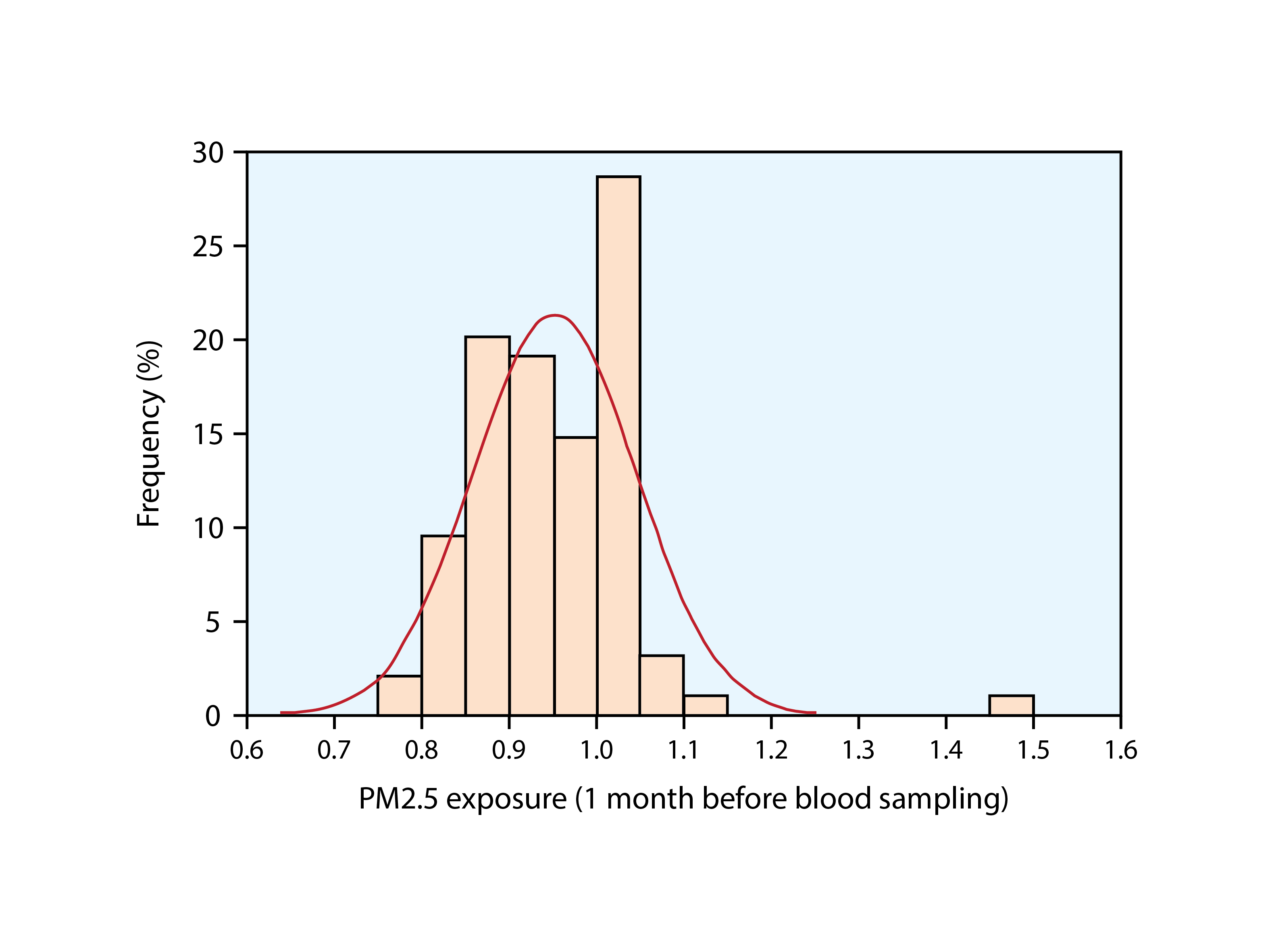

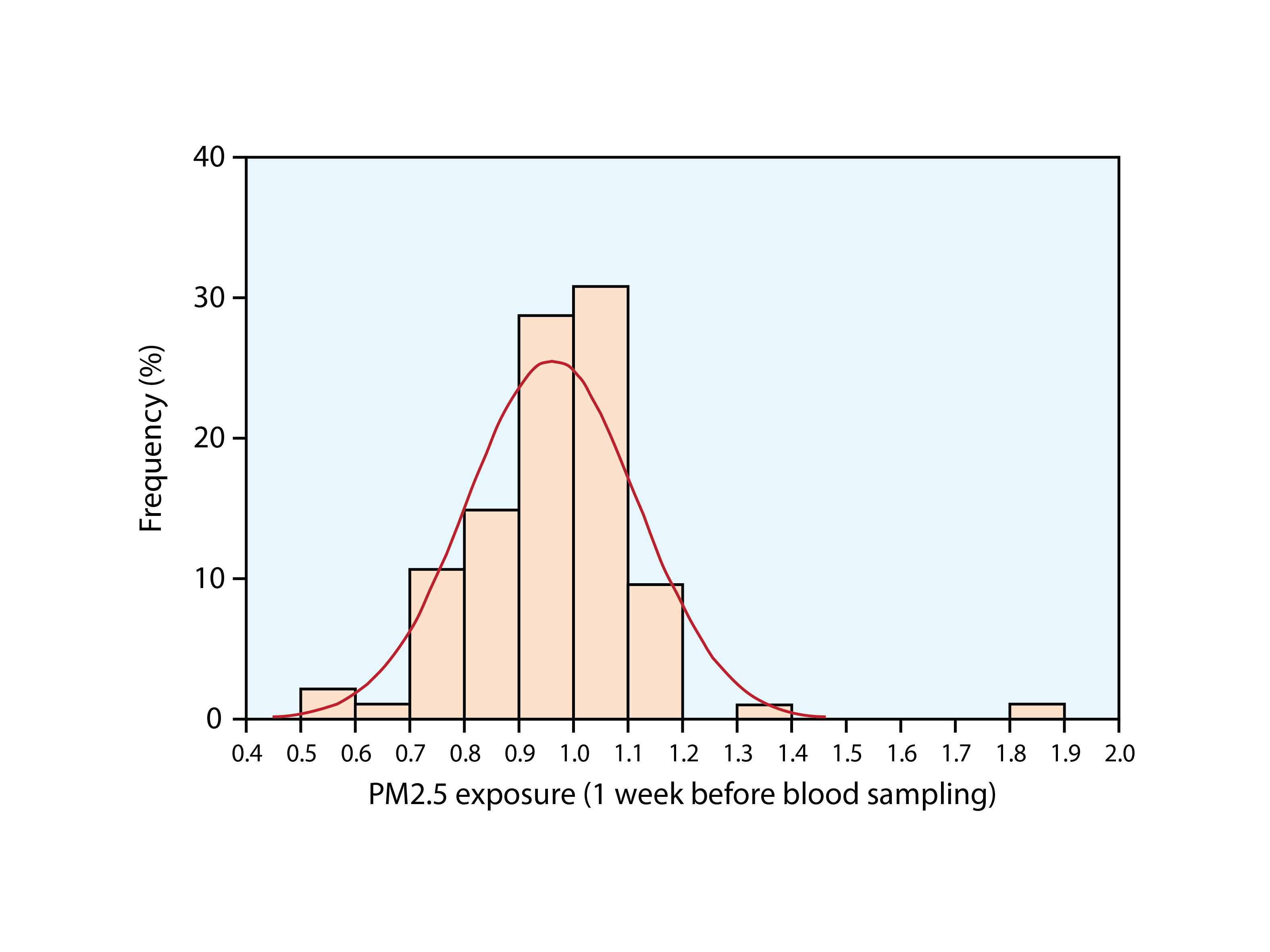
**

b)

a)

**Figure s1**. Distribution of PM_2.5_ exposure averaged for 1 month (a) and 1 week (b) prior to blood draw. Units are µg/m^3^

**Figure s2**

**
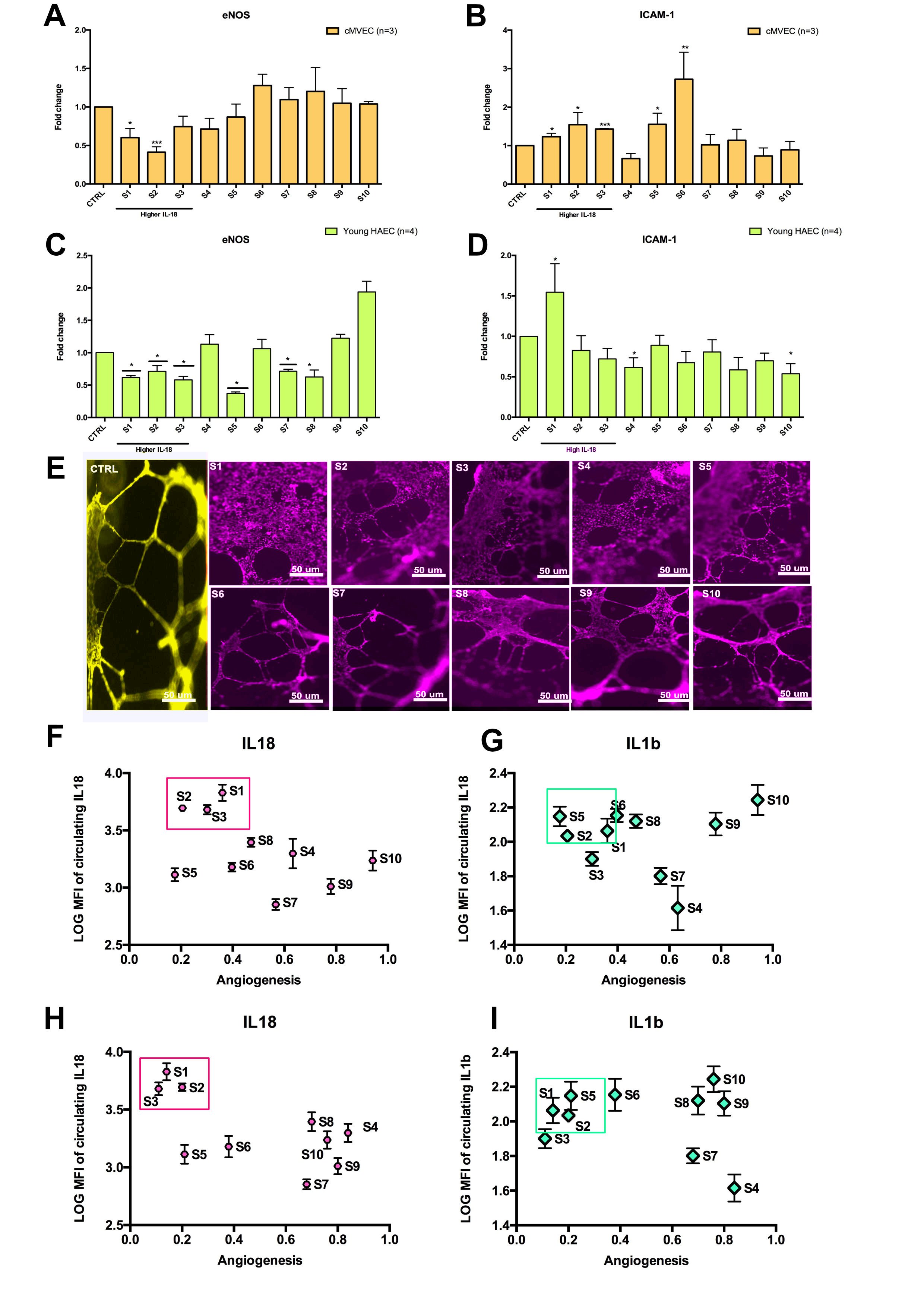
**

**Figure s2.** Characterization of endothelial damage under plasma of exposed to PM_2.5_**.** To characterize endothelial cell dysfunction under plasmatic stress, we starved the cells for 24h (1% serum) and exposed them with 25% of plasma for 24 h in EGM2 medium. Plasma were from 10 participants that had been exposed to a spectrum of PM_2.5_ levels. To determine if there was an endothelial cell origin specificity, we evaluated eNOS **(A)** and ICAM-1 **(B)** by qRT-PCR on cMVEC (n=3) from 50 year-old donors and to evaluate any age-mediated-effect, we used HAEC (n=4) from 30-year-old donors (**C, D, E**)**.** Next, we evaluated if there was any association between IL-18 **(F)** or IL1-β **(G)** plasma levels from these 10 participants and angiogenesis dysfunction in cMVEC and HAEC from young donors (**H, I**). Values are presented as means ± SEM.

**Figure s3**

**
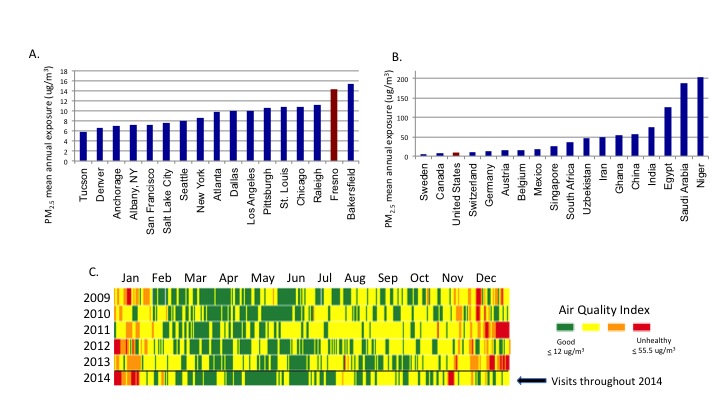
**


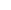


**Figure s3.** Heat map of PM_2.5_ levels versus time are plotted for the duration of the study (2014) and previous 5 years with air quality index (AQI) categories. Data and heatmap obtained from EPA.gov. Month (number of subject visits): Jan(1), Feb(0), March (2), April (20), May (7), June (12), July (14), August (13), September (8), October (20), November (2), December (1).
